# Searching for the universality of nudging: A cross-cultural comparison of the information effects of reminding people about familial support

**DOI:** 10.1371/journal.pone.0277969

**Published:** 2022-11-22

**Authors:** Hidenori Komatsu, Hiromi Kubota, Nobuyuki Tanaka, Hirotada Ohashi, Mariah Griffin, Jennifer Link, Glenn Geher, Maryanne L. Fisher

**Affiliations:** 1 Grid Innovation Research Laboratory, Central Research Institute of Electric Power Industry, Yokosuka, Kanagawa, Japan; 2 Sustainable System Research Laboratory, Central Research Institute of Electric Power Industry, Abiko, Chiba, Japan; 3 School of Engineering, The University of Tokyo, Bunkyo, Tokyo, Japan; 4 Department of Psychology, State University of New York at New Paltz, New Paltz, New York, United States of America; 5 Animal Behavior Graduate Group, University of California, Davis, Davis, California, United States of America; 6 Department of Psychology, Faculty of Science, Saint Mary’s University, Halifax, Nova Scotia, Canada; OsloMet - storbyuniversitetet, NORWAY

## Abstract

Nudging is a method for eliciting a desired behavior. One approach to nudging involves information provision. When information presented for this purpose is designed from an evolutionary perspective, it may reveal a deeper level of rationality within human decision-making that might otherwise appear to be irrational. Based on insights from the evolution of altruism, we previously designed a message to remind people of the benefits they have received from the actions of relatives to realize industrialization. We then demonstrated that using this message in Japan was effective at moderating extreme risk-averse attitudes toward air pollution resulting from industrialization. However, the universality of the intervention effect, including whether it could be affected by exogenous factors, was not explored. Therefore, in the present study, we conducted a randomized controlled trial based on an online survey carried out in Japan, Canada, and the US. The intervention was shown to be effective in all the three countries, but the effect size varied according to segment. Although women showed more intervention effects than men in Japan and the US, no significant sex difference was observed in Canada. In terms of personality traits, higher agreeableness significantly contributed to the intervention effects. The influence of the COVID-19 pandemic, which necessitated many lifestyle changes, was found to weaken the intervention effect by increasing the message effect in the control group. We propose that this effect was caused by an increased perception of familial support in everyday life. These results suggest that the nudge message was universally effective, although the effect size might have been affected by cultural factors and social events.

## Introduction

Thaler and Sunstein popularized the concept of nudging, defining it as “any aspect of the choice architecture that alters people’s behavior in a predictable way without forbidding any options or significantly changing their economic incentives” [1]. A nudge stimulates intuitive decision-making and leads people to make better choices. A wide range of schemes involving nudges has been proposed in various domains [2]. Although nudging relies on the irrationality of human decision-making, an examination from the viewpoint of evolutionary psychology might reveal rationality at a deeper level within human decision-making, which has long been considered anomalous [3]. However, to date, few nudges have been designed based on insights from evolutionary psychology. Establishing such a framework would be useful for designing nudges more efficiently by minimizing the trial-and-error experiments that usually accompany costly randomized control trials and require large numbers of samples [4]. Thus, the use of an evolutionary perspective might contribute to a more economical methodology for designing information provision to elicit intuitive responses based on human evolution [3, 5].

Among the many theories based on evolutionary principles, we focused on the evolution of altruism, which is considered to be universal in humans [6, 7], and hence could be a basis for effective interventions. There are many well-known theories that explain altruism, including kin selection [8] and reciprocal altruism based on direct reciprocity [9–11] or indirect reciprocity [12, 13]. For this variety of altruism, several possible interpretations have been also proposed. One possibility relies on the assumption that altruistic behaviors also function as signaling for courtship displays that serve as a mating strategy [14]. Another interpretation contains the view that selection pressures work on multi-levels, where cooperation can evolve if the selection pressure is stronger at the group-level than at the individual-level. This concept, so-called ‘multi-level selection’, is essentially compatible with kin selection to explain the evolutionary adaptiveness of altruism [15]. In the meantime, for the intersection of an evolutionary perspective and risk attitude, a significant body of evidence exists that indicates attitudes toward risks are domain-specific and thereby depend on situations [16–18]. However, domain-generality of risk attitudes [19], which is not necessarily at odds with the domain-specificity, has not been totally rejected and thus further research is required [20].

Although the abovementioned studies analyzing risk attitude from evolutionary perspectives have attracted much attention, there has been no study that has explored which factor rooted in altruistic evolution can affect risk attitude. Thus, in our previous work, we proposed an evolutionary multi-agent simulation model based on the assumption that the subjective assessment of a source of risk may be influenced by altruism due to kin selection when the risk is perceived to endanger future generations [21]. Although it is phenomenologically known that risk perception is influenced by whether the risk will affect future generations [22], how such responses are formed is still unclear. Furthermore, extreme attitudes toward risks are an important political science issue [23, 24] and such attitudes sometimes lead to the damage of public goods. For example, public opinion moved the government to establish regulations to reduce dioxin emissions in Japan. Although dioxin was originally perceived to be highly dangerous, the toxicity was actually much lower and the cost-effectiveness of the regulations was determined to be undesirable [25]. Public funds should instead be used for more cost-effective countermeasures in terms of saving lives—in other words, for maximizing public goods.

Against this backdrop, we developed a new approach to nudging that elicits altruistic behavior based on a sense of familial support that moderates an extreme aversion toward risks perceived to endanger future generations [26]. The concept of this form of nudging was based on insight obtained from the evolutionary multi-agent simulation model that we previously developed. Agents evolved to be risk-averse when the environment was moderate while they evolved to be risk-prone, on average, when the environment is harsh, and within the population, agents who received more support from their relatives always evolved to be more risk-prone independent of the environmental situation. Using this insight from the simulation model, we designed and delivered information about air pollution to moderate risk-averse attitudes toward industrialization, as a case study. Our designed message based on the insights from our evolutionary multi-agent simulation model, which highlighted support from previous generations to promote sense of being supported, successfully moderated risk-averse attitudes in the real world too [26].

Although we demonstrated that the intervention effect of the nudge message was significant in Japan, whether this type of nudging is universally effective or has different effects per segment in other parts of the world is unclear. Given that agreeableness, which could be a superordinate trait of altruism, is universal to humans [27] and that psychological responses rooted in evolutionary adaptation are context-sensitive [28], we can assume that nudges eliciting altruistic responses might be universally effective, even if the degree of the effect varies depending on other factors such as culture.

In this work, we carried out similar experiments in other countries, using messages to remind questionnaire respondents of the benefits they have received from their ancestors that manifest in daily life. Building on our previous survey results, we performed a cross-cultural comparison to investigate the universality of the nudge messages among people living in Japan, Canada, and the US. We chose these two North American countries because of their cultural diversity and high percentage of immigrants [29], which allows for an appropriate comparison with Japan and its relatively homogenous society and low percentage of immigrants.

## Materials and methods

### Survey overview

We conducted an online survey to investigate the intervention effects of providing information as a nudge. Although we designed the experimental questionnaires, we outsourced their distribution and collection to an online survey company. Respondents were individuals who had registered with the company to participate in online surveys. The company obtained written informed consent from all the participants on our behalf. The survey was anonymized and did not collect any personal information. Furthermore, no biological samples were obtained from the respondents, and they were assumed not to be subjected to any psychological distress as a result of their participation. The surveys were approved by the relevant ethics committees of the Central Research Institute of Electric Power Industry in Japan and Saint Mary’s University in Canada. For the State University of New York at New Paltz’s contributions, data collection was not conducted as part of the work of that particular team. As such, their contributions were deemed as “non-human subjects research” in line with the policies of the university’s Human Research Ethics Board. This study was conducted in accordance with the Declaration of Helsinki and its later amendments.

Our analysis was based on four datasets (Table 1): samples obtained on February 28 and March 1, 2019 in Japan (J-2019), samples obtained from February 25 to 27, 2020 in Japan (J-2020), samples obtained from February 27 to March 12, 2020 in Canada (C-2020), and samples obtained from February 27 to March 12, 2020 in the US (U-2020). For all four datasets, the respondents were aged 20 years or older, which means that the study did not include any minors. Each respondent’s characteristics, including age and sex, were obtained from the survey company’s records. Each dataset consisted of equal numbers of male and female respondents.

**Table 1:** Basic attributes of the 4 datasets.

Dataset		J-2019	J-2020	C-2020	U-2020
Survey term		Feb. 28–Mar. 1, 2019	Feb. 25–27, 2020	Feb. 27–Mar. 12, 2020	Feb. 27–Mar. 12, 2020
Number of responses	Men	2,021	2,067	2,064	2,065
	Women	2,041	2,063	2,063	2,063
	Total	4,062	4,130	4,127	4,128

The questionnaires were available in English, French, and Japanese. Respondents in Japan could choose only the Japanese version, respondents in the US could choose only the English version, and respondents in Canada could choose either the English or the French versions. In each country, the survey was offered in the national language(s) of that country.

Pathogens are known to change people’s various distinct responses, including their attitude toward politics [30] and environmental problems [31]. The “finite pool of worry hypothesis” [32, 33] suggests that people’s focus on a risk can be overridden by another larger risk because people’s total amount of worry is limited. Considering that COVID-19 has become a worldwide health concern since its onset, the COVID-19 pandemic is highly likely to have altered the nudging effects of our designed messages. Although we used three datasets from 2020 (J-2020, C-2020, and U-2020) to investigate the universality of the intervention, the intervention effects seem to have been moderated by the COVID-19 pandemic in only the J-2020 dataset. Thus, we included J-2019, which was collected before the COVID-19 pandemic for reference because the intervention effect was clearer than the one in J-2020. The cumulative numbers of reported cases of COVID-19 closest to the start of the survey term for each country [34–36] are shown in Table 2. By including J-2019 in the analysis, we also explored how the COVID-19 pandemic affected the intervention effects. We collected samples from different respondents in J-2019 and J-2020, meaning that the respondents in all four datasets received our nudge messages only once.

**Table 2:** Cumulative number of reported cases of COVID-19 (Japan: Feb. 24–Mar. 1, 2020; Canada: Feb. 27, 2020; US: Feb. 27, 2020).

Country	Prefecture/Province/State	Count
Japan	Kanagawa	503
	Tokyo	60
	Hokkaido	45
	Aichi	28
	Kyoto	26
	Chiba	21
	Wakayama	13
	Ishikawa	6
	Saitama, Osaka, Kumamoto	4
	Nara, Fukuoka	3
	Nagano, Gifu, Kagoshima	2
	Miyagi, Tochigi, Niigata, Shizuoka, Mie, Tottori, Ehime, Okinawa	1
Canada	British Columbia	7
	Ontario	6
US	California	33
	Oklahoma	19
	Illinois	2
	Arizona, Massachusetts, Washington	1

**Fig 1:**
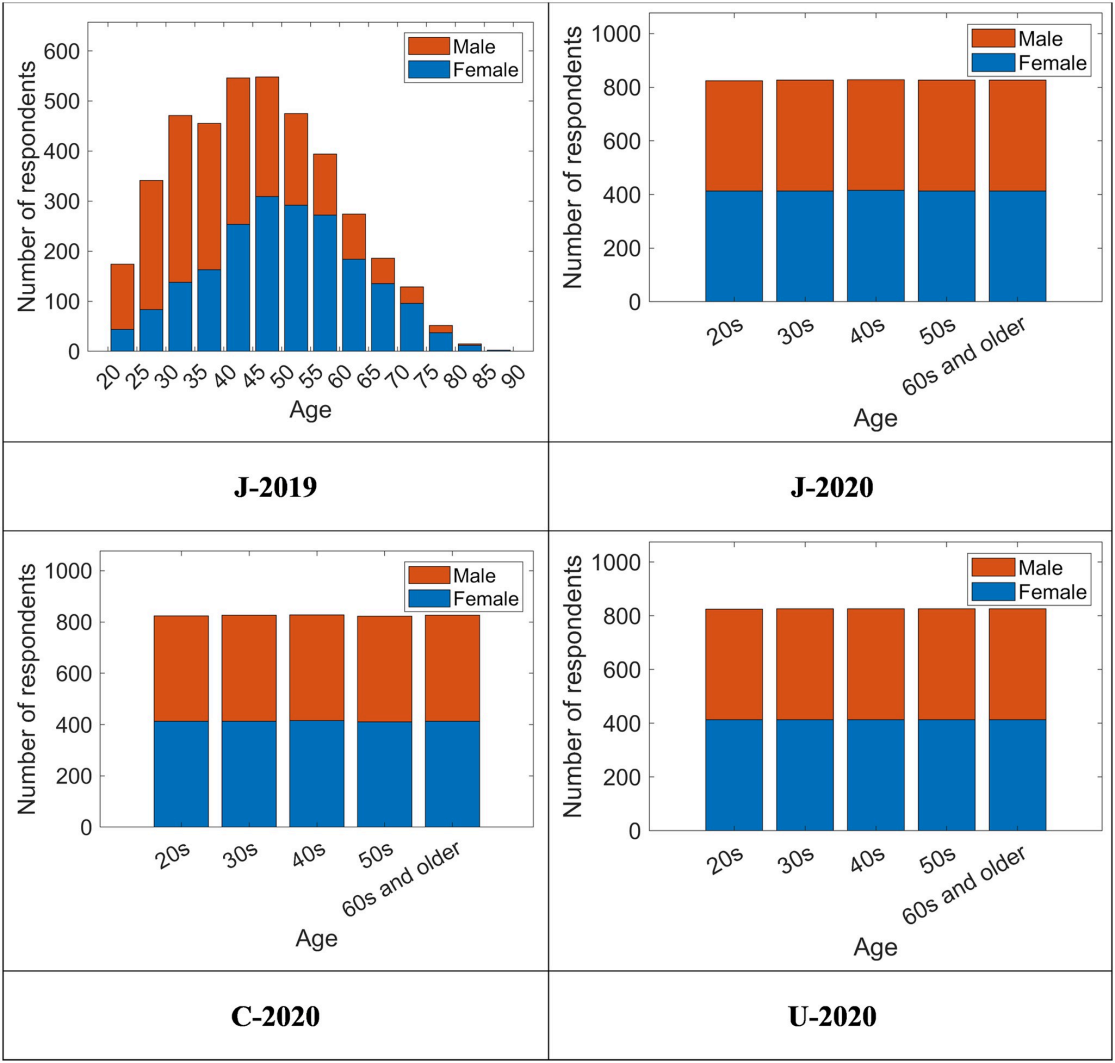
Number of respondents by age and sex.

We excluded respondents who reported having more children living with them, or more children working in paid jobs, than their number of children. The total number of valid samples for each dataset and sex ratios are shown in Table 1. Although responses were collected based on the frequency distribution of age groups in J-2019, the three datasets from 2020 were collected with an equal number of responses in the age groups of 20s, 30s, 40s, 50s, and 60s and older (Fig 1). All other conditions were identical across the four datasets.

### Backgrounds in common

The three countries in our survey have different cultures. Nevertheless, they have some similarities in their basic backgrounds. Here we introduce these similarities, which do not have explanatory power for the cultural differences and hence are not directly used for further analysis.

The averages of agreeableness, which is related to altruism (and of interest to the present work) are 49.9 for Canada, 49.1 for the US, and 48.8 for Japan [37]. Given that the values range from 46.1 to 54.2 across 51 different cultures [37], our three target countries can be considered relatively similar in terms of agreeableness.

The respondent’s number of children, how many children were living with the respondents, and how many children were working but still living at home are shown in Table 3. Although the US had the largest numbers of children among the three countries, the differences were relatively small. The numbers of respondents who lived with their parents and who were working are shown in Table 4. Canada and the US had larger numbers of each compared with Japan, but the differences were again relatively small.

**Table 3:** Characteristics of respondents’ children.

Dataset		J-2019	J-2020	C-2020	U-2020
**Number of children**		**Counts**			
	0	1,733 (42.7%)	1,941 (47.0%)	1,876 (45.5%)	1,692 (41.0%)
	1	724 (17.9%)	657 (15.9%)	752 (18.2%)	722 (17.5%)
	2	1,183 (29.2%)	1,146 (27.7%)	915 (22.2%)	977 (23.7%)
	3	364 (9.0%)	332 (8.0%)	403 (9.8%)	442 (10.7%)
	4	39 (1.0%)	46 (1.1%)	116 (2.8%)	179 (4.3%)
	5 or more	13 (0.3%)	8 (0.2%)	65 (1.6%)	116 (2.8%)
**Mean number**		1.1	1.0	1.1	1.4
**Number of children living with respondent**					
	0	2,244 (55.3%)	2,571 (62.3%)	2,675 (64.8%)	2,579 (62.5%)
	1	868 (21.4%)	800 (19.4%)	732 (17.7%)	738 (17.9%)
	2	753 (18.5%)	613 (14.8%)	524 (12.7%)	546 (13.2%)
	3	175 (4.3%)	132 (3.2%)	150 (3.6%)	185 (4.5%)
	4	17 (0.4%)	12 (0.3%)	33 (0.8%)	63 (1.5%)
	5 or more	4 (0.1%)	2 (0.0%)	13 (0.3%)	17 (0.4%)
**Mean number**		0.8	0.6	0.6	0.8
**Number of children who are working**					
	0	3,105 (76.4%)	3,053 (73.9%)	2,951 (71.5%)	2,858 (69.2%)
	1	438 (10.8%)	450 (10.9%)	489 (11.8%)	521 (12.6%)
	2	393 (9.7%)	483 (11.7%)	451 (10.9%)	474 (11.5%)
	3	112 (2.8%)	129 (3.1%)	169 (4.1%)	180 (4.4%)
	4	10 (0.2%)	14 (0.3%)	48 (1.2%)	50 (1.2%)
	5 or more	4 (0.1%)	1 (0.0%)	19 (0.5%)	45 (1.1%)
**Mean number**		0.4	0.5	0.5	0.7

**Table 4:** Characteristics of respondents’ parents.

Dataset	J-2019	J-2020	C-2020	U-2020
**Living with parents in the same house or at the same site**	**Counts**			
With neither parent	3,163 (77.9%)	3,095 (74.9%)	3,433 (83.2%)	3,415 (82.7%)
With either parent	373 (9.2%)	376 (9.1%)	312 (7.6%)	412 (10.0%)
With both parents	526 (13.0%)	659 (16.0%)	382 (9.3%)	301 (7.3%)
**Parents working in paid jobs**				
Neither parent	2,709 (66.7%)	2,632 (63.7%)	2,802 (67.9%)	2,840 (68.8%)
Either parent	739 (18.2%)	770 (18.6%)	672 (16.3%)	740 (17.9%)
Both parents	614 (15.1%)	728 (17.6%)	653 (15.8%)	548 (13.3%)

The quality of outdoor air in each country may potentially influence the message effects because our designed messages mention air pollution. The Environmental Protection Agency in the US defined the standard for outdoor PM2.5 concentration as an annual mean of 12 μg/m^3^, labeling air quality as “Good” if the level is lower than the standard [38]. The population-weighted average levels of exposure to PM2.5 for Japan, Canada, and the US have been below this standard since 2016 [39]. Thus, the outdoor air in all three target countries is similar and adequately clean.

### Statistical analysis

In all the analyses, we set two explained variables: perceived danger of air pollution resulting from industrialization to future generations (Future Generations) and that to respondents themselves (Yourself), where the former is our main target variable. Responses were obtained by using the question shown in S1 Questionnaire in S1 File. We first performed an analysis of variance (ANOVA) and found that the attitudes toward the risks were homogenous within the same explained variable, but that the perceived danger to Future Generations was larger than that to Yourself, using responses before receiving the designed messages. We then estimated the intervention effects of our nudge message by using a difference-in-difference (DID) estimation, in which the effect was defined as differences of the effect size between a control group and the treatment groups. The intervention effects were also investigated using a correlation analysis of the perception of benefiting from the actions of older relatives. Next, we compared the message effects per segment to determine how effect sizes differed by country, sex, and age. Statistical significance of the differences across our designed groups or segments were calculated using the Wilcoxon signed-rank test. All error bars in graphs were computed as 95% confidence intervals based on t-distributions. Finally, we performed panel analysis using forced-entry linear regression. All of these analyses were performed using Matlab R2019b with Statistical and Machine Learning Toolbox Ver. 11.6.

### Survey design

**Fig 2:**
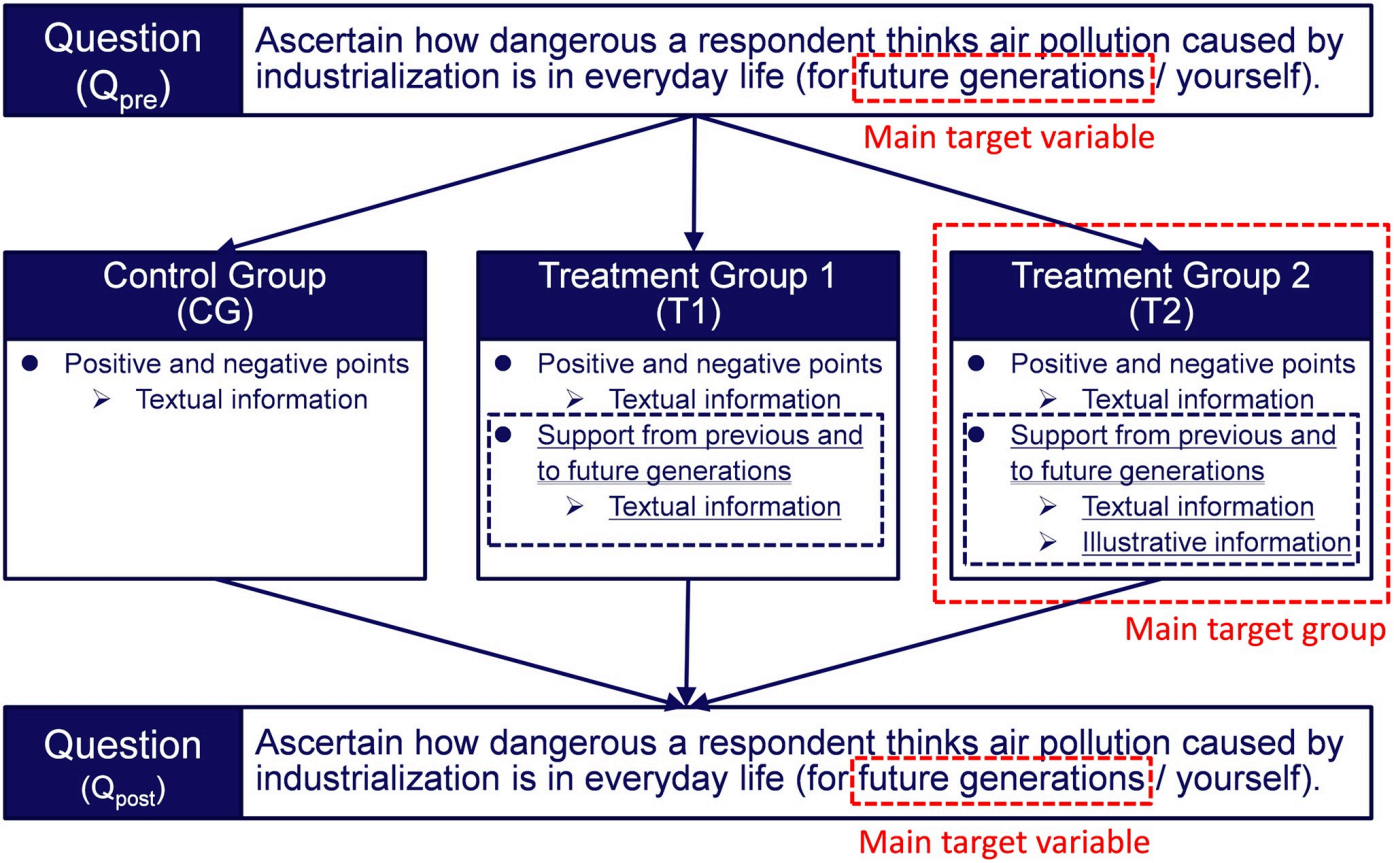
Diagram of the experimental procedure. The interventions are indicated by black dashed boxes and the targets of those interventions are indicated by red dashed boxes. Reprinted from [26] under a CC BY license, with permission from Komatsu, 2020.

The survey design has been described previously [26]. The figures in this section are reproduced from our previous report. Briefly, a randomized controlled trial involving an online survey was conducted to investigate whether the nudge messages could moderate negative attitudes toward air pollution. Fig 2 is a flowchart of the experimental procedure.

**Fig 3:**
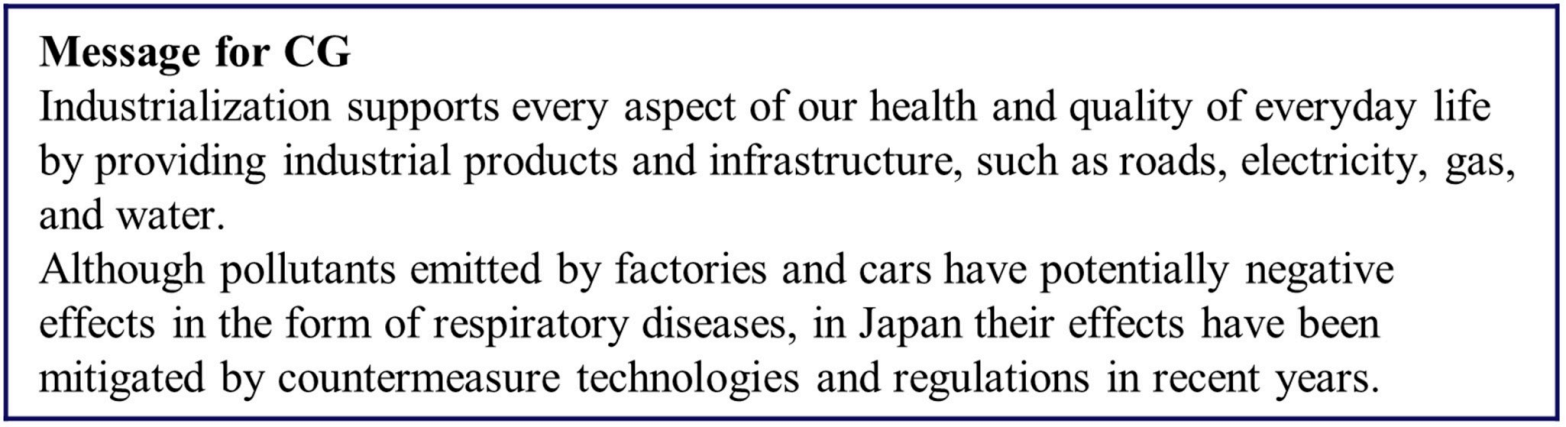
Message presented to the control group. Reprinted from [26] under a CC BY license, with permission from Komatsu, 2020.

**Fig 4:**
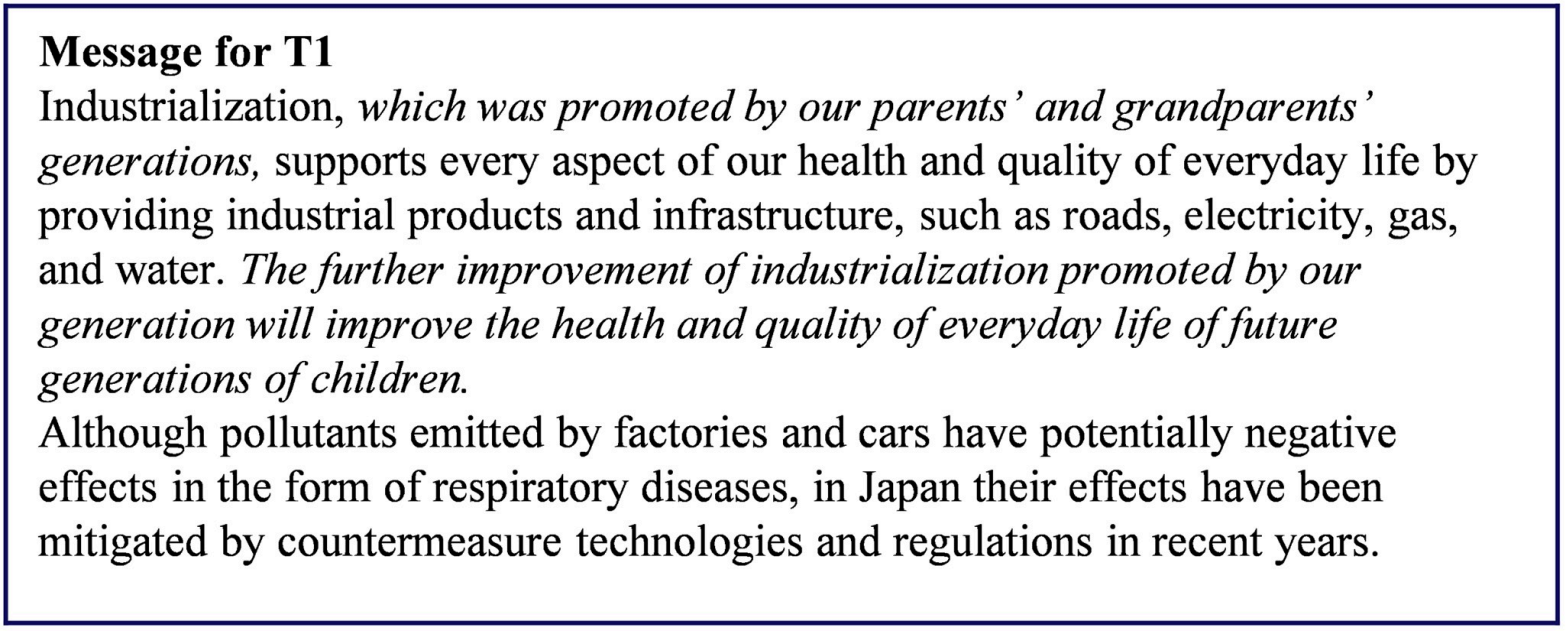
Message presented to treatment group 1. The intervention portions are indicated by italics. Reprinted from [26] under a CC BY license, with permission from Komatsu, 2020.

**Fig 5:**
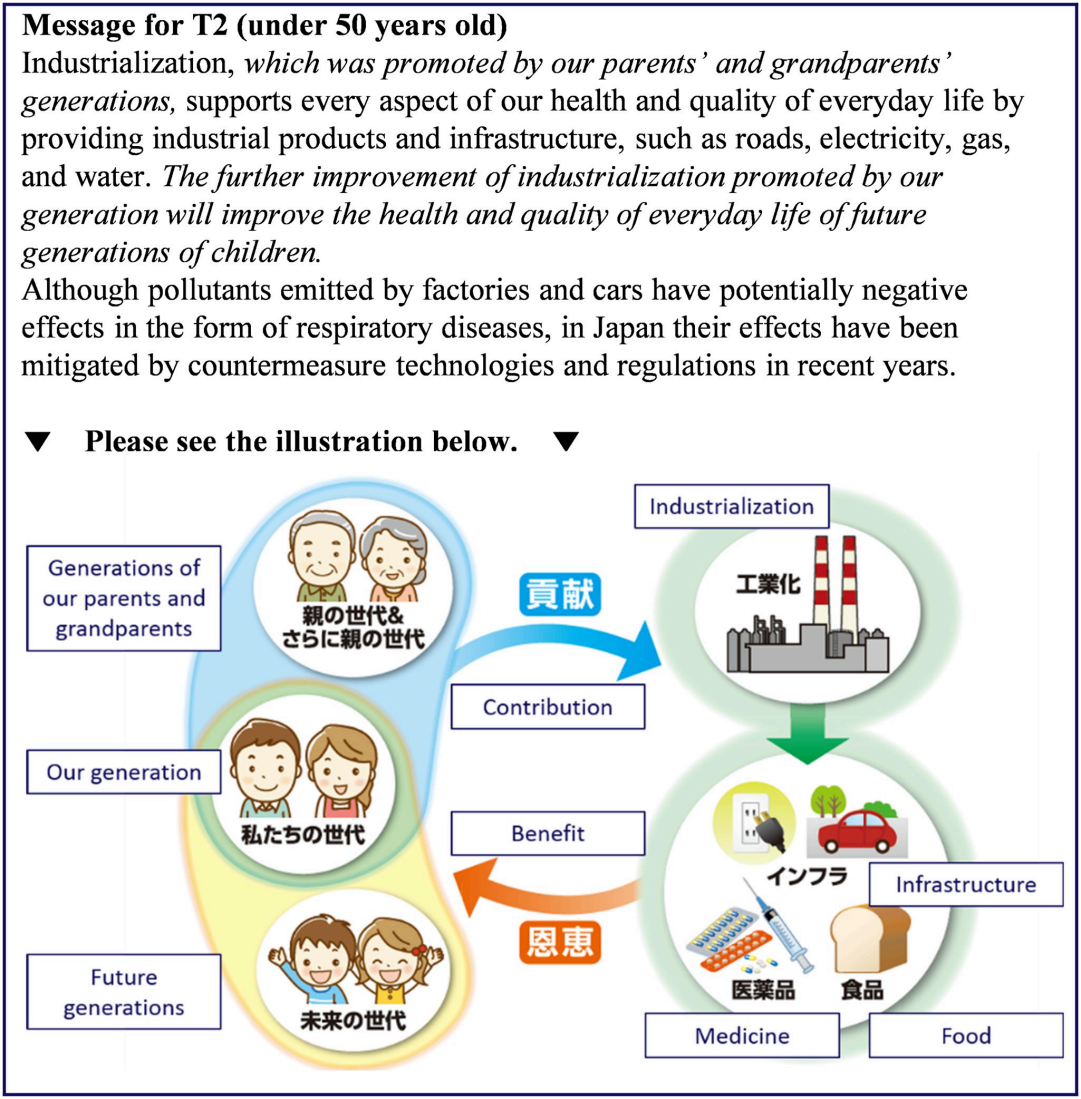
Message presented to those under 50 years of age in treatment group 2. The intervention portions are indicated by italics. Reprinted from [26] under a CC BY license, with permission from Komatsu, 2020.

**Fig 6:**
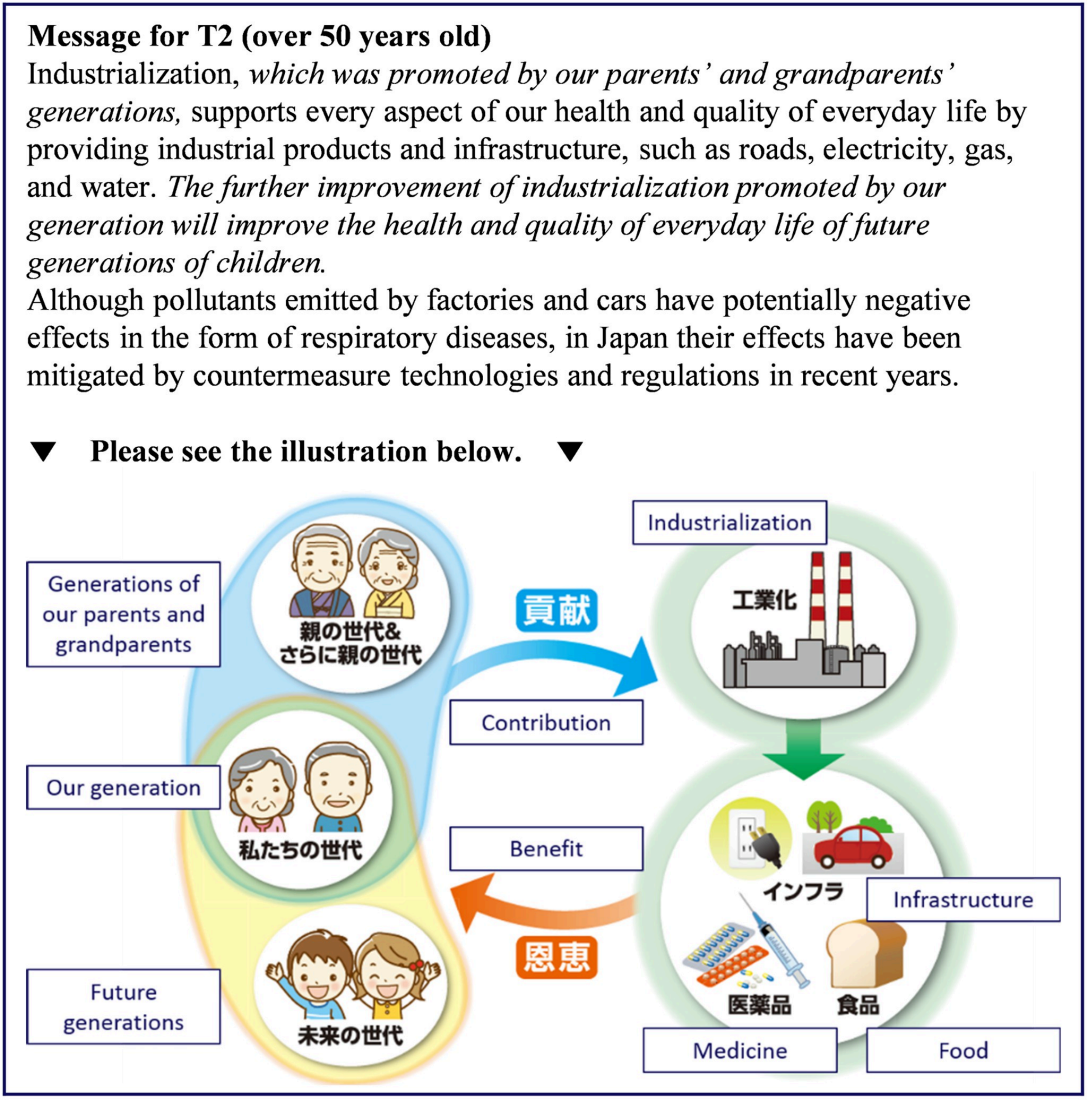
Message presented to those 50 years of age and older in treatment group 2. The intervention portions are indicated by italics. Reprinted from [26] under a CC BY license, with permission from Komatsu, 2020.

Respondents were first asked to consider the danger posed by air pollution resulting from industrialization to the daily lives (*Q*_pre_) of future generations as well as to themselves. The responses were measured on 5-point Likert-type scales (1 = Safe, 3 = Neutral, 5 = Dangerous). Next, the respondents were randomly assigned one of the following groups: a control group (CG), which received information about the risks of air pollution and the benefits of industrialization; treatment group 1 (T1), which received a nudge in the form of a message about the benefits realized from the actions of their ancestors; and treatment group 2 (T2), which received the same message as T1 plus an illustration highlighting its content. The intervention for T2 differed slightly for respondents under the age of 50 and those 50 years of age and older. The messages presented to the participants are shown in Figs 3–6 and a summary of the characteristics of the presented information is given in Table 5.

**Table 5:** Summary of experimental interventions.

Information provided	Presentation type	CG	T1	T2 (Main target group)
Positive and negative aspects of industrialization	Text	✔	✔	✔
Support from previous generations / to future generations	Text		✔	✔
	Illustration			✔
**No. of samples**	J-2019	1,358	1,354	1,350
	J-2020	1,378	1,376	1,376
	C-2020	1,375	1,375	1,377
	U-2020	1,375	1,377	1,376

CG, control group; T1, treatment group 1; T2, treatment group 2. The check mark (✔) indicates that the information was provided.

CG was given a passage of text about the positive and negative aspects of industrialization and was designed to be relatively neutral. The positive points included the benefits conferred by industrial products and infrastructure, while the negative points included air pollutants and their potential to cause respiratory diseases. It was noted that air pollution has been decreasing in recent years.

T1 was given the same information that CG received plus two additional sentences. The additional text suggested that the present generation benefited from the actions of previous generations to realize industrialization, and that future generations will similarly benefit from the additional efforts of one’s own generation. These additional messages were expected to enhance the sense of receiving support from previous generations.

T2 was given the same information and message as T1 plus an illustration highlighting how previous generations contributed to industrialization, as well as the benefits to one’s own generation and future generations in the form of industrial products. In consideration of the wide range of participant age groups, the illustrations of one’s own generation and previous generations were changed according to age group (under 50 years or 50 years and older). We presented older people in the traditional clothing that their parents and grandparents wore because those over 50 years old were less likely to have living parents. After presenting the messages, we again asked the respondents in all three groups to answer the same question they were shown before the intervention (*Q*_post_) on 5-point Likert-type scales (1 = Safe, 3 = Neutral, 5 = Dangerous), which is the same as *Q*_pre_. After *Q*_post_, the respondents were asked other relevant questions shown in ‘Other questions’ of Supplementary Information in the order as they are listed. Finally, Big Five personality traits were measured by TIPI-J [40], which is a Japanese version of the Ten Item Personality Inventory [41]. These questions used for the survey except for TIPI-J are described in S1 Questionnaire in S1 File.

Although there are two opposing views toward air pollution given the costs versus benefits of industrialization, for the analysis, we first focus on moderating extreme risk-averse attitudes, which sometimes damage public goods, as a political science issue. Then, we analyze how our designed messages might also increase the perceived benefits.

For more natural and inclusive wording, we chose “parents’ and grandparents’ generations” and “future generations of children” in the designed messages to remind respondents about familial support. These might be relatively indirect expressions compared with wording indicating the respondents’ own parents or children. However, in our ex-post analysis, we investigated how these expressions actually increased the respondents’ perceived support from older relatives, as well as that given to younger relatives, and then considered the appropriate nudging effects.

## Results

### Before interventions

**Fig 7:**
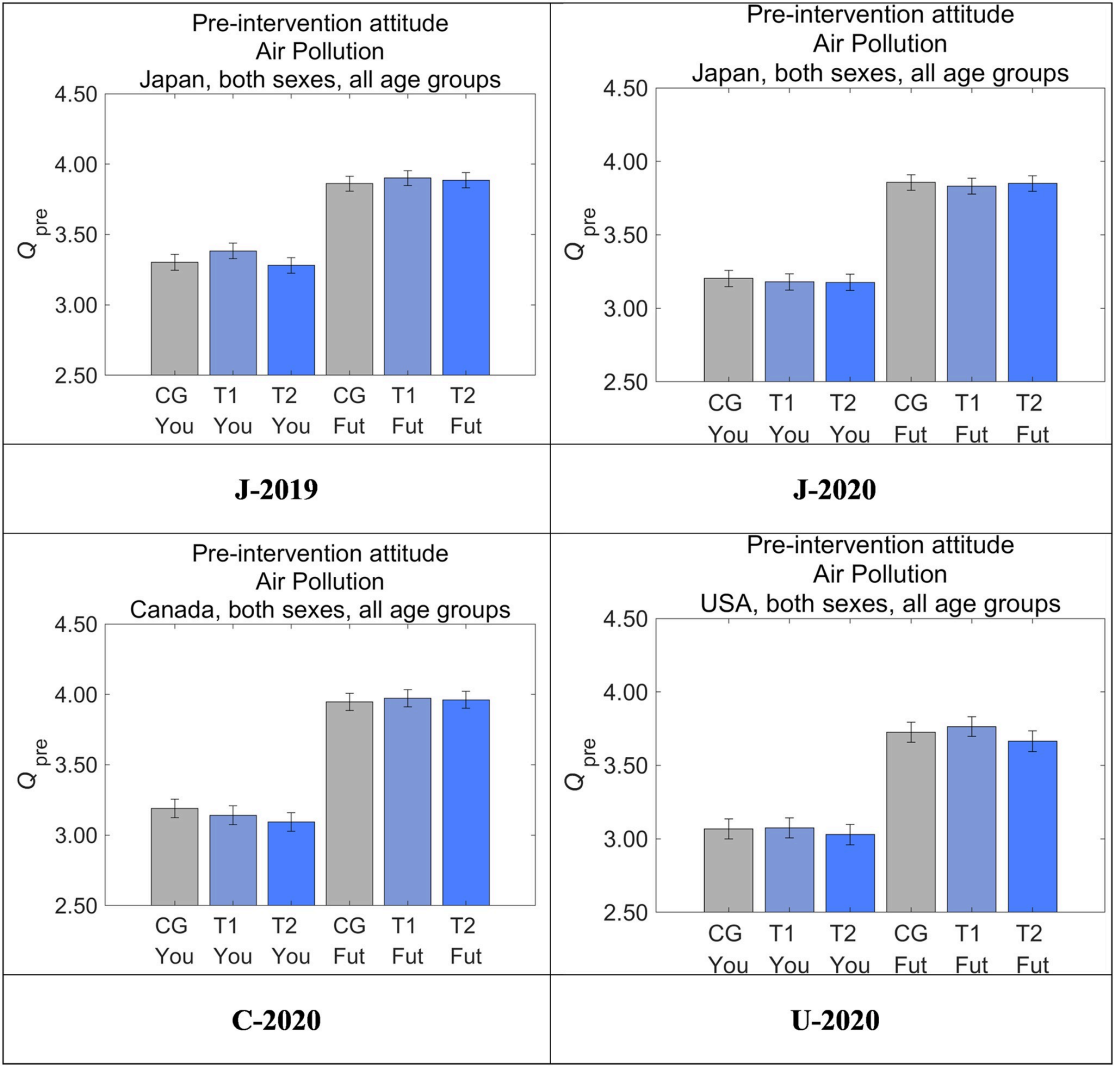
Pre-intervention attitude toward air pollution by group. Higher values on the vertical axis indicate higher perceived danger of air pollution. Error bars show 95% confidence intervals.

The attitudes of respondents toward the effects of air pollution on Future Generations and Yourself before the interventions (responses to *Q*_pre_) are shown for each group in Fig 7. We performed an ANOVA to compare attitudes toward Future Generations (Tables 6 and 7) and Yourself (Tables 8 and 9). There were no significant differences among the three groups except between T1 and T2 in J-2019 for attitudes toward their own generation (*p* < 0.05). We did not find any bias in the respondent attributes that might cause this difference, which may be considered an incidental realization within the controlled randomization, suggesting that the samples were sufficiently randomized. This kind of initial difference in perceptions is adjusted in our evaluation of the intervention effects by using a DID estimation, to compare the changes in attitudes caused by information provision.

**Table 6:** One-way analysis of variance for pre-intervention (*Q*_pre_) attitudes toward air pollution for future generations (Future Generations).

Dataset	Source	SS	df	MS	F	Probability > F
J-2019	Group	1.113	2	0.556	0.551	0.576
	Error	4098.638	4059	1.010		
	Total	4099.751	4061			
J-2020	Group	0.457	2	0.229	0.223	0.800
	Error	4240.294	4127	1.027		
	Total	4240.751	4129			
C-2020	Group	0.449	2	0.224	0.171	0.843
	Error	5425.034	4124	1.315		
	Total	5425.483	4126			
U-2020	Group	6.956	2	3.478	2.080	0.125
	Error	6897.259	4125	1.672		
	Total	6904.215	4127			

SS, sum of squares; df, degrees of freedom; MS, mean squares; F, F-ratio.

**Table 7:** Comparison of pre-intervention (*Q*_pre_) attitudes toward air pollution for future generations (Future Generations).

Dataset	Compared groups		Upper end of 95% CI	Estimated mean	Lower end of 95% CI	*p*
J-2019	T1	CG	0.131	0.040	−0.050	0.550
	T2	CG	0.115	0.024	−0.066	0.803
	T2	T1	0.075	−0.016	−0.106	0.911
J-2020	T1	CG	0.066	−0.025	−0.115	0.795
	T2	CG	0.084	−0.007	−0.097	0.983
	T2	T1	0.109	0.018	−0.072	0.885
C-2020	T1	CG	0.128	0.025	−0.077	0.830
	T2	CG	0.117	0.015	−0.088	0.940
	T2	T1	0.092	−0.011	−0.113	0.967
U-2020	T1	CG	0.154	0.039	−0.077	0.710
	T2	CG	0.055	−0.061	−0.176	0.433
	T2	T1	0.016	−0.100	−0.215	0.107

95% CI, 95% confidence interval; CG, control group; T1, treatment group 1; T2, treatment group 2.

**Table 8:** One-way analysis of variance for pre-intervention (*Q*_pre_) attitudes toward air pollution for the respondents themselves (Yourself).

Dataset	Source	SS	df	MS	F	Probability > F
J-2019	Group	7.896	2	3.948	3.618	0.027
	Error	4429.272	4059	1.091		
	Total	4437.168	4061			
J-2020	Group	0.586	2	0.293	0.272	0.762
	Error	4449.854	4127	1.078		
	Total	4450.441	4129			
C-2020	Group	6.263	2	3.131	2.023	0.132
	Error	6382.380	4124	1.548		
	Total	6388.643	4126			
U-2020	Group	1.630	2	0.815	0.476	0.621
	Error	7057.991	4125	1.711		
	Total	7059.622	4127			

SS, sum-of-squares; df, degrees of freedom; MS, mean squares; F, F-ratio.

**Table 9:** Comparison of pre-intervention (*Q*_pre_) attitudes toward air pollution for the respondents themselves (Yourself).

Dataset	Compared groups		Upper end of 95% CI	Estimated mean	Lower end of 95% CI	*p*
J-2019	T1	CG	0.175	0.081	−0.013	0.110
	T2	CG	0.072	−0.022	−0.116	0.849
	T2	T1	-0.008	−0.103	−0.197	0.029
J-2020	T1	CG	0.069	−0.024	−0.116	0.821
	T2	CG	0.066	−0.027	−0.119	0.780
	T2	T1	0.090	−0.003	−0.096	0.997
C-2020	T1	CG	0.063	−0.048	−0.159	0.569
	T2	CG	0.016	−0.095	−0.207	0.109
	T2	T1	0.064	−0.047	−0.159	0.577
U-2020	T1	CG	0.123	0.006	−0.110	0.991
	T2	CG	0.078	−0.039	−0.155	0.719
	T2	T1	0.072	−0.045	−0.162	0.639

95% CI, 95% confidence interval; CG, control group; T1, treatment group 1; T2, treatment group 2.

The value for Future Generations was consistently higher than that for Yourself regardless of the group, suggesting that the effect of air pollution is considered more dangerous to Future Generations than to Yourself. Compared with Canada and Japan, the US had a tendency to regard air pollution as less dangerous, suggesting that Americans may be relatively less concerned about air pollution compared with Japanese and Canadians.

Although a direct comparison between J-2019 and J-2020 is not ideal given the different age distribution of the samples, the pre-intervention attitudes toward air pollution showed a tendency toward moderation in J-2020 for both Yourself and Future Generations, despite the influence of the COVID-19 pandemic that was already starting to spread in Japan during the J-2020 survey term.

### Overall intervention effects

**Fig 8:**
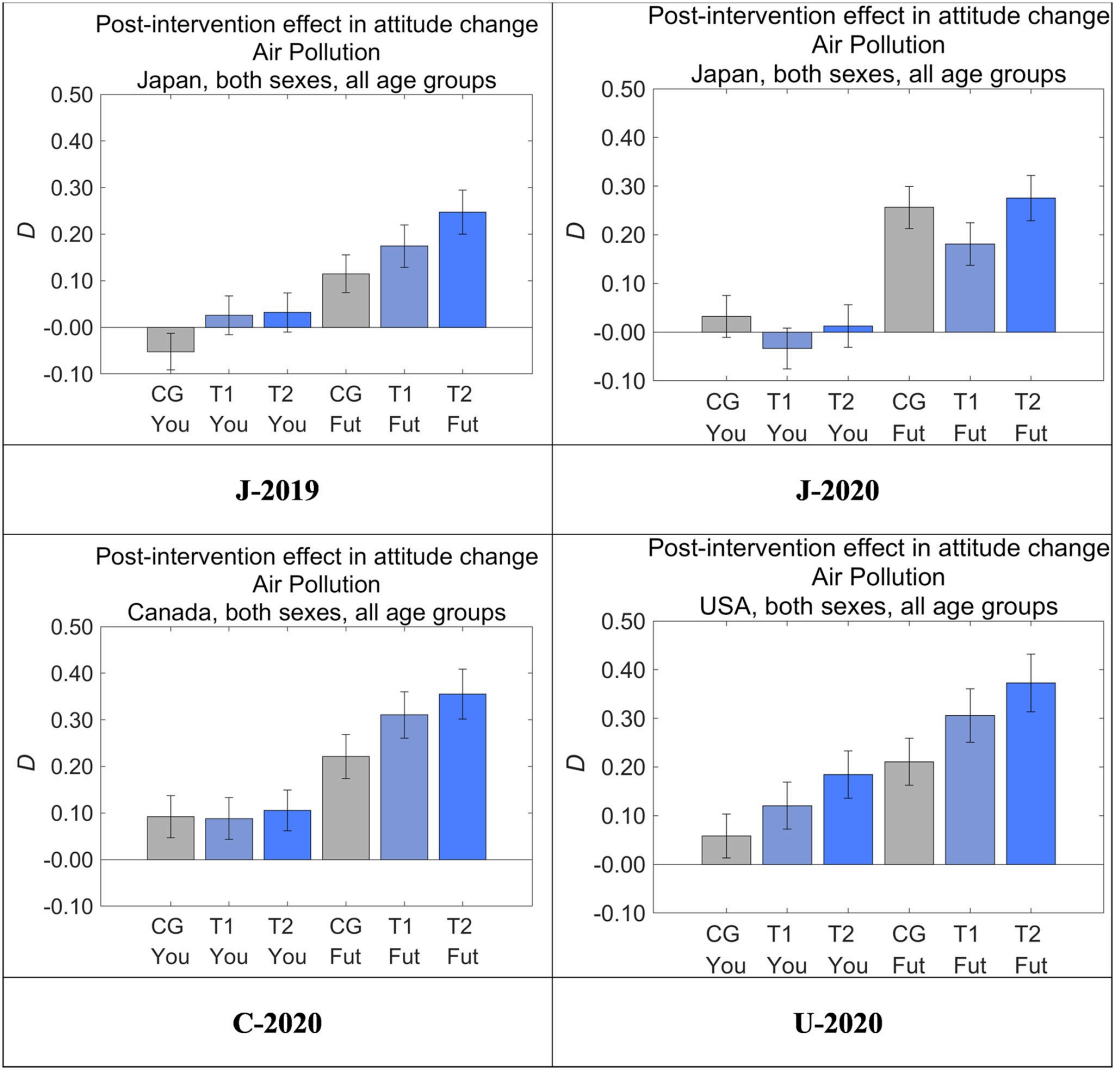
Post-intervention effect in attitude change toward air pollution (*D*). Higher values on the vertical axis indicate lower perceived danger of air pollution. Error bars show 95% confidence intervals.

The degree of post-intervention in attitude change toward air pollution was defined as the difference between the answers to *Q*_post_ and *Q*_pre_ in each sample (*D*). Fig 8 shows *D* for Yourself and Future Generations according to dataset and message group. *D* was positive for all groups in J-2019 (but not J-2020, see below), C-2020, and U-2020 for Future Generations, with the highest averages found in T2, followed by T1 and then CG. *D* for T2 was larger than that for CG (*p* < 0.001) in all three of these datasets. These results suggest that pairing the illustration with the additional text in T2 increased *D* compared with CG, and the illustration in T2 also increased *D* compared with T1 in all three countries in the absence of the COVID-19 pandemic. Through this cross-cultural comparison, we found that our nudge messages had significant intervention effects that were independent of country; that is, the message effects for moderating perceived threats to future generations were consistently T2 > T1 > CG in all the three countries, suggesting the universality of the message effects. Although even the T1 effects showed a clear increase compared with CG, the T2 message should be practically employed because T2 showed larger effect sizes than T1.

We also found that the COVID-19 pandemic had an impact on the intervention effect by increasing the message effect in the control groups rather than decreasing the effect in the treatment groups. In J-2020, which was collected when the COVID-19 pandemic was starting to spread in Japan, the intervention effect of T2 compared with CG (i.e., the DID effect) was not significant, unlike in the other three datasets. The decreased DID effect in T2 of J-2020 might have been affected by COVID-19, which had just begun spreading in Japan at the start of the survey period.

Focusing on the intervention effects for Yourself, the DID effects were significantly positive in J-2019 and U-2020. The effect size is especially large in U-2020, where the positive *D* for CG was further increased by T2, whereas the negative *D* for CG was made positive by T2 in J-2019. *D* for CG in J-2020 was increased compared with J-2019 and eventually decreased the DID effect of T2 in J-2020, which was the same effect as for Future Generations. There were no significant differences in *D* for Yourself among CG, T1, and T2 in C-2020. These tendencies suggest that the intervention effects for Yourself are weaker and not as universally effective as for Future Generations.

To clarify the statistical significance of all combinations of the three message groups, we also performed ANOVAs to compare the attitude change (*D*) toward Future Generations (Tables 10 and 11) as well as Yourself (Tables 12 and 13). Again, the target DID effects were the differences in T2 and CG for Future Generations, all of which were significantly positive except for J-2020 (Table 11). The effects for Yourself were significantly positive only for J-2019 and U-2020 (Table 13).

**Table 10:** One-way analysis of variance for post-intervention effect in attitude change toward air pollution (*D*) for future generations (Future Generations).

Dataset	Source	SS	df	MS	F	Probability > F
J-2019	Group	11.931	2	5.966	8.586	0.000
	Error	2820.311	4059	0.695		
	Total	2832.242	4061			
J-2020	Group	6.859	2	3.430	4.813	0.008
	Error	2941.123	4127	0.713		
	Total	2947.983	4129			
C-2020	Group	12.819	2	6.409	7.089	0.001
	Error	3728.532	4124	0.904		
	Total	3741.351	4126			
U-2020	Group	18.205	2	9.103	8.595	0.000
	Error	4368.864	4125	1.059		
	Total	4387.070	4127			

SS, sum of squares; df, degrees of freedom; MS, mean squares; F, F-ratio.

**Table 11:** Comparison of post-intervention effect in attitude change toward air pollution (*D*) for future generations (Future Generations).

Dataset	Compared groups		Upper end of 95% CI	Estimated mean	Lower end of 95% CI	*p*
J-2019	T1	CG	0.134	0.059	−0.016	0.152
	T2	CG	0.208	0.133	0.057	0.000
	T2	T1	0.148	0.073	−0.002	0.059
J-2020	T1	CG	0.000	−0.075	−0.151	0.051
	T2	CG	0.095	0.019	−0.056	0.821
	T2	T1	0.170	0.094	0.019	0.009
C-2020	T1	CG	0.174	0.089	0.004	0.036
	T2	CG	0.219	0.134	0.049	0.001
	T2	T1	0.130	0.045	−0.040	0.436
U-2020	T1	CG	0.187	0.095	0.003	0.041
	T2	CG	0.254	0.162	0.070	0.000
	T2	T1	0.159	0.067	−0.025	0.201

95% CI, 95% confidence interval; CG, control group; T1, treatment group 1; T2, treatment group 2.

**Table 12:** One-way analysis of variance for post-intervention effect in attitude change toward air pollution (*D*) for the respondents themselves (Yourself).

Dataset	Source	SS	df	MS	F	Probability > F
J-2019	Group	5.974	2	2.987	5.037	0.007
	Error	2407.014	4059	0.593		
	Total	2412.988	4061			
J-2020	Group	3.098	2	1.549	2.366	0.094
	Error	2701.847	4127	0.655		
	Total	2704.946	4129			
C-2020	Group	0.223	2	0.111	0.158	0.854
	Error	2917.353	4124	0.707		
	Total	2917.576	4126			
U-2020	Group	10.991	2	5.495	6.860	0.001
	Error	3304.447	4125	0.801		
	Total	3315.438	4127			

SS, sum-of-squares; df, degrees of freedom; MS, mean squares; F, F-ratio.

**Table 13:** Comparison of post-intervention effect in attitude change toward air pollution (*D*) for the respondents themselves (Yourself).

Dataset	Compared groups		Upper end of 95% CI	Estimated mean	Lower end of 95% CI	*p*
J-2019	T1	CG	0.147	0.078	0.009	0.022
	T2	CG	0.153	0.084	0.015	0.012
	T2	T1	0.075	0.006	−0.063	0.978
J-2020	T1	CG	0.007	−0.065	−0.138	0.086
	T2	CG	0.053	−0.020	−0.092	0.801
	T2	T1	0.118	0.046	−0.027	0.298
C-2020	T1	CG	0.071	−0.004	−0.080	0.990
	T2	CG	0.088	0.013	−0.062	0.914
	T2	T1	0.092	0.017	−0.058	0.852
U-2020	T1	CG	0.142	0.062	−0.018	0.160
	T2	CG	0.206	0.126	0.046	0.001
	T2	T1	0.144	0.064	−0.016	0.145

95% CI, 95% confidence interval; CG, control group; T1, treatment group 1; T2, treatment group 2.

**Fig 9:**
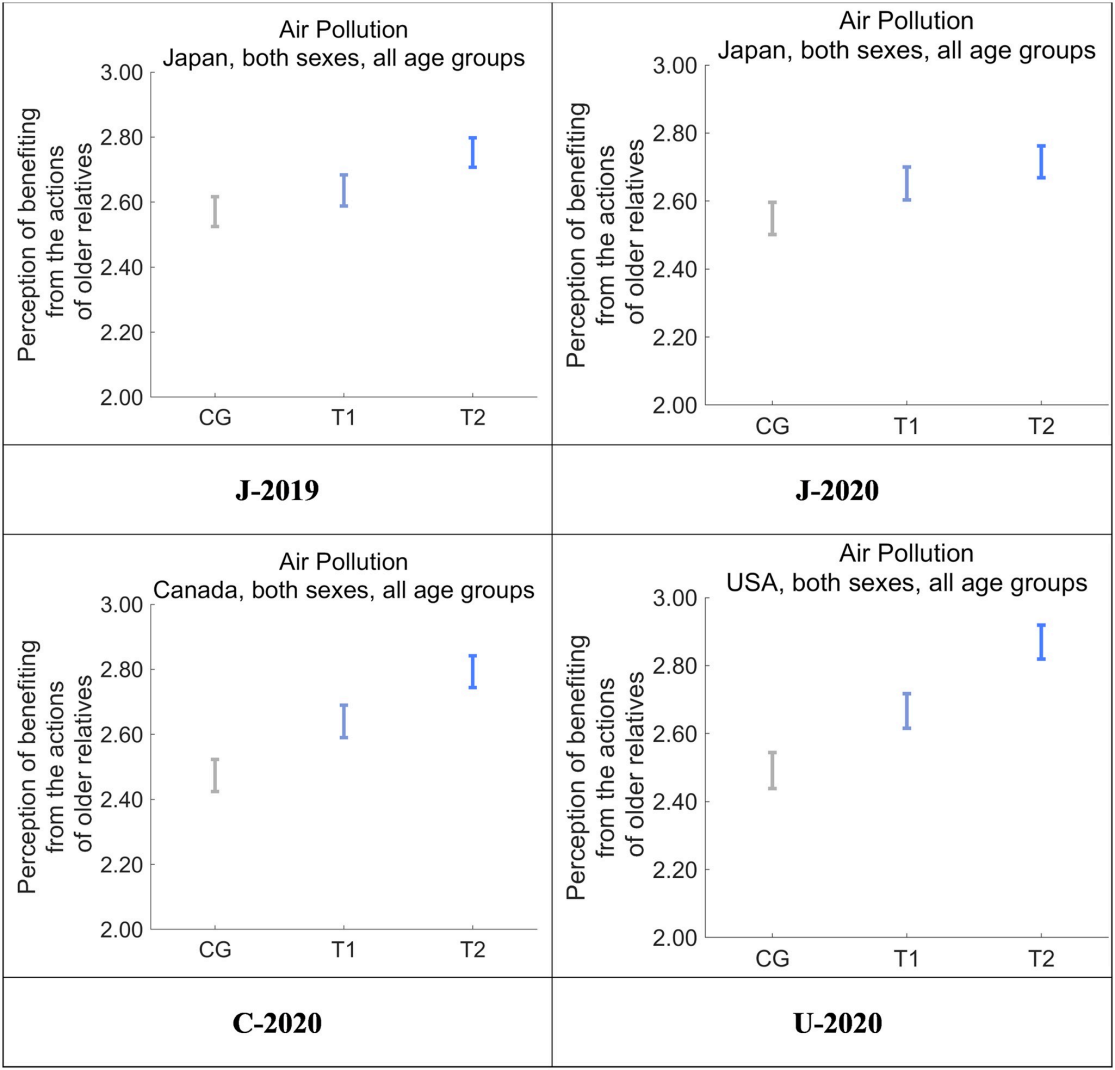
Perception that the respondent has benefited from the actions of relatives belonging to older generations to realize industrialization after receiving one of the nudge message interventions. Error bars show 95% confidence intervals.

**Fig 10:**
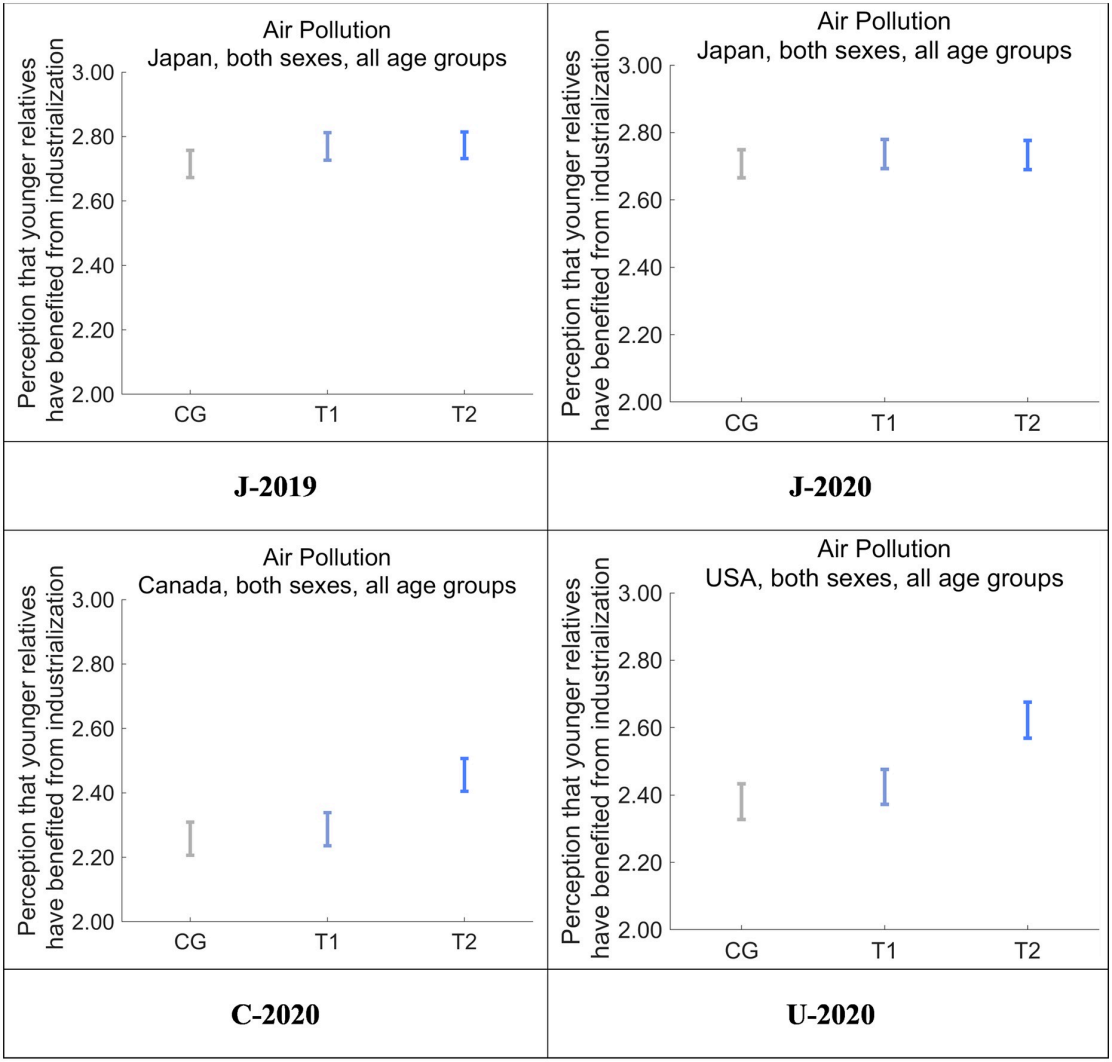
Perception that the respondents’ younger relatives have benefited from industrialization after receiving one of the nudge message interventions. Error bars show 95% confidence intervals.

Next, we asked respondents to rate the degree to which they feel that their health and quality of life are the result of actions taken by relatives belonging to previous generations, including their parents and grandparents (Fig 9). Perceived benefits were highest in T2, followed by T1 and CG in all four datasets, and T2 was significantly higher than CG (*p* < 0.001). The differences were clearer in Canada and the US compared with Japan. We then asked respondents to rate the degree to which they feel that industrialization facilitates the health and quality of life for their younger relatives, including their children and grandchildren (Fig 10). Perceived benefits were highest in T2, followed by T1 and CG in C-2020 and U-2020, and the differences between CG and T2 were significant (*p* < 0.001). Compared with these results, the differences among CG, T1, and T2 were not significant in J-2019 and J-2020, suggesting that the intervention in T2 universally increased the perception of benefiting from the actions of one’s older relatives (e.g., parents, grandparents) more than the perception that one’s younger relatives (e.g., children, grandchildren) are similarly benefiting.

To investigate how the nudge message intervention that moderates negative attitudes toward air pollution is related to the perception of benefiting from the actions of older relatives and the perception that younger relatives are similarly benefiting, we performed a correlation analysis for each of the four datasets (Tables 14 and 15). Aggregating the samples from all three message groups, *D* for both one’s own generation and future generations was significantly correlated with the perception of benefiting from the actions of older relatives and the perception that younger relatives are similarly benefiting in all datasets.

**Table 14:** Correlation coefficients between post-intervention effect in attitude change (*D*) and perceptions of benefiting from the actions of previous generations.

Dataset	J-2019		J-2020		C-2020		U-2020	
Group	Future Generations	Yourself	Future Generations	Yourself	Future Generations	Yourself	Future Generations	Yourself
**CG + T1 + T2**	***0.068	***0.085	***0.089	***0.084	***0.108	***0.102	***0.089	***0.098
**CG**	0.032	0.038	0.041	0.026	**0.083	***0.091	0.035	^†^0.044
**T1**	**0.087	***0.111	***0.106	***0.113	***0.113	***0.117	***0.097	***0.132
**T2**	**0.067	***0.095	***0.119	***0.117	***0.108	***0.099	***0.103	***0.094

CG, control group; T1, treatment group 1; T2, treatment group 2.

^†^, *, **, *** Difference from zero with 90%, 95%, 99%, and 99.9% confidence, respectively.

**Table 15:** Correlation coefficients between post-intervention effect in attitude change (*D*) and perceptions that younger relatives are benefiting from the actions of previous generations.

Dataset	J-2019		J-2020		C-2020		U-2020	
Group	Future Generations	Yourself	Future Generations	Yourself	Future Generations	Yourself	Future Generations	Yourself
**CG + T1 + T2**	***0.070	***0.071	***0.066	*0.034	***0.162	***0.109	***0.133	***0.090
**CG**	**0.085	***0.091	0.039	0.021	***0.165	***0.125	***0.099	*0.063
**T1**	*0.064	^†^0.051	**0.081	0.028	***0.167	***0.098	***0.106	*0.066
**T2**	*0.058	*0.068	**0.077	*0.054	***0.146	***0.103	***0.173	***0.124

CG, control group; T1, treatment group 1; T2, treatment group 2.

^†^, *, **, *** Difference from zero with 90%, 95%, 99%, and 99.9% confidence, respectively.

In T1 and T2, the perception of benefiting from the actions of older relatives was significant for both Yourself and Future Generations. Given that the four datasets except for C-2020 did not show significant correlations in CG (*p* < 0.01 for Future Generations and *p* < 0.001 for Yourself, respectively), the nudge message interventions for T1 and T2 appear to have successfully increased the perception of benefit. These results can be corresponded to the findings obtained from the evolutionary simulation model we previously developed, which suggested that risk-averse attitudes could be moderated when people perceived that they were supported by relatives [21]. Although T1 and T2 values for Future Generations were significantly correlated with the perception that younger relatives are benefiting, the correlations with Yourself were weaker than with Future Generations. These results suggest that when the perception of benefiting from the actions of older relatives and the perception that younger relatives are similarly benefiting are increased, the feeling that air pollution is dangerous could be moderated. Furthermore, for the perceptions that younger generations are benefiting from the actions of previous generations, the correlations were larger for Future Generations than Yourself.

Regardless of the moderated DID effect in J-2020 compared with J-2019, there were no clear post-intervention differences in the perceptions of benefiting from the actions of older relatives and the perceptions that younger relatives are similarly benefiting. Considering the increased *D* in CG in J-2020, where no correlations with either perception were observed for Future Generations and Yourself, the potential factor might be an increased sense of benefiting in everyday life.

**Fig 11:**
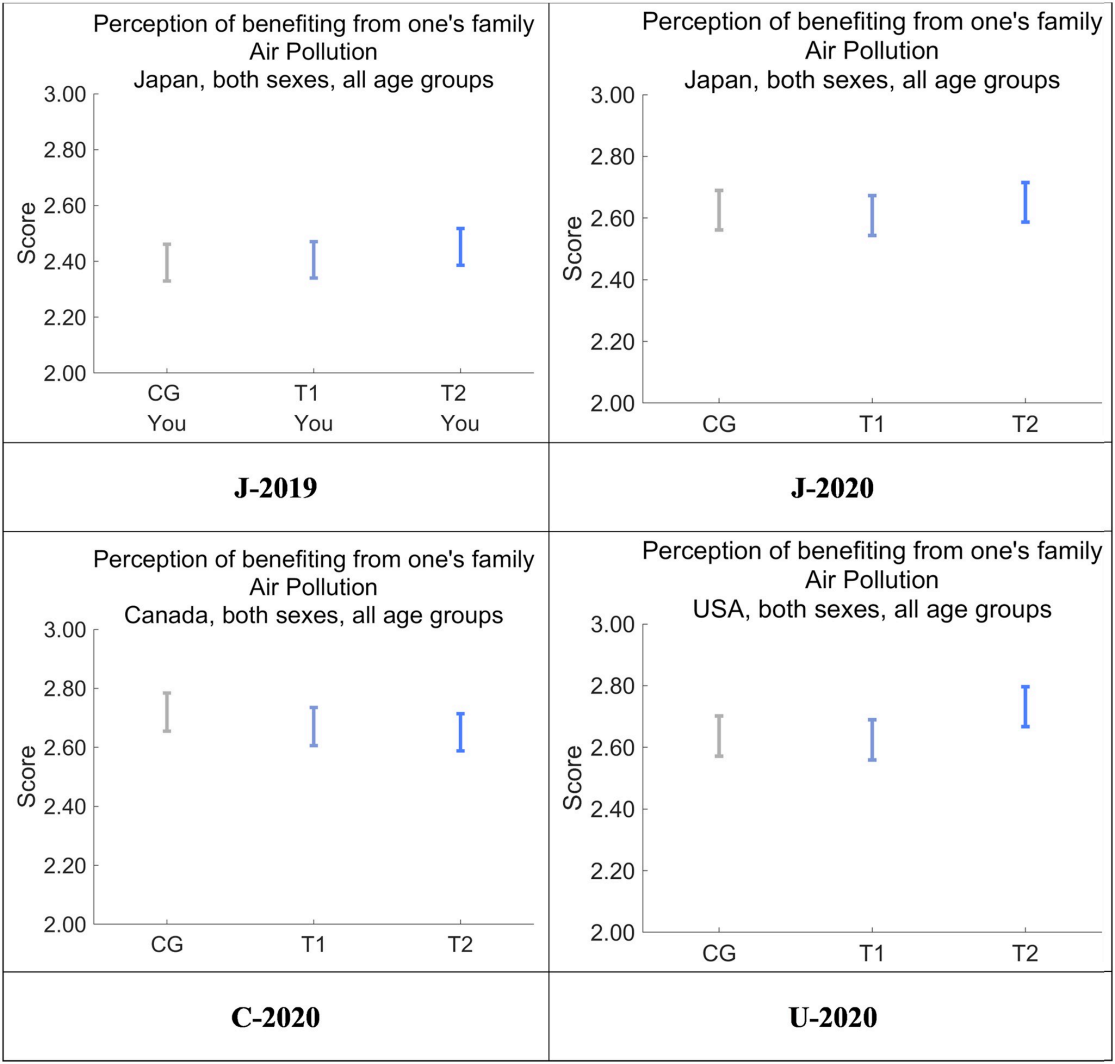
Perception that the respondent is benefiting from family in everyday life. Error bars show 95% confidence intervals.

Fig 11 shows the perception of benefiting from family in everyday life in all four datasets. The perceptions were significantly increased in J-2020 compared with J-2019 in all message groups (*p* < 0.001), suggesting that the COVID-19 pandemic in Japan may have increased the sense of familial support in everyday life, although the designed nudge messages themselves did not affect this sense of support. It is noteworthy that the perception of familial support in everyday life reached the same degree as in C-2020 and U-2020 only after the COVID-19 pandemic started to spread in Japan. This finding is consistent with the fact that *D* in CG for Future Generations is especially low in J-2019 compared with the other three datasets (Fig 8).

### Intervention effects by segment

**Fig 12:**
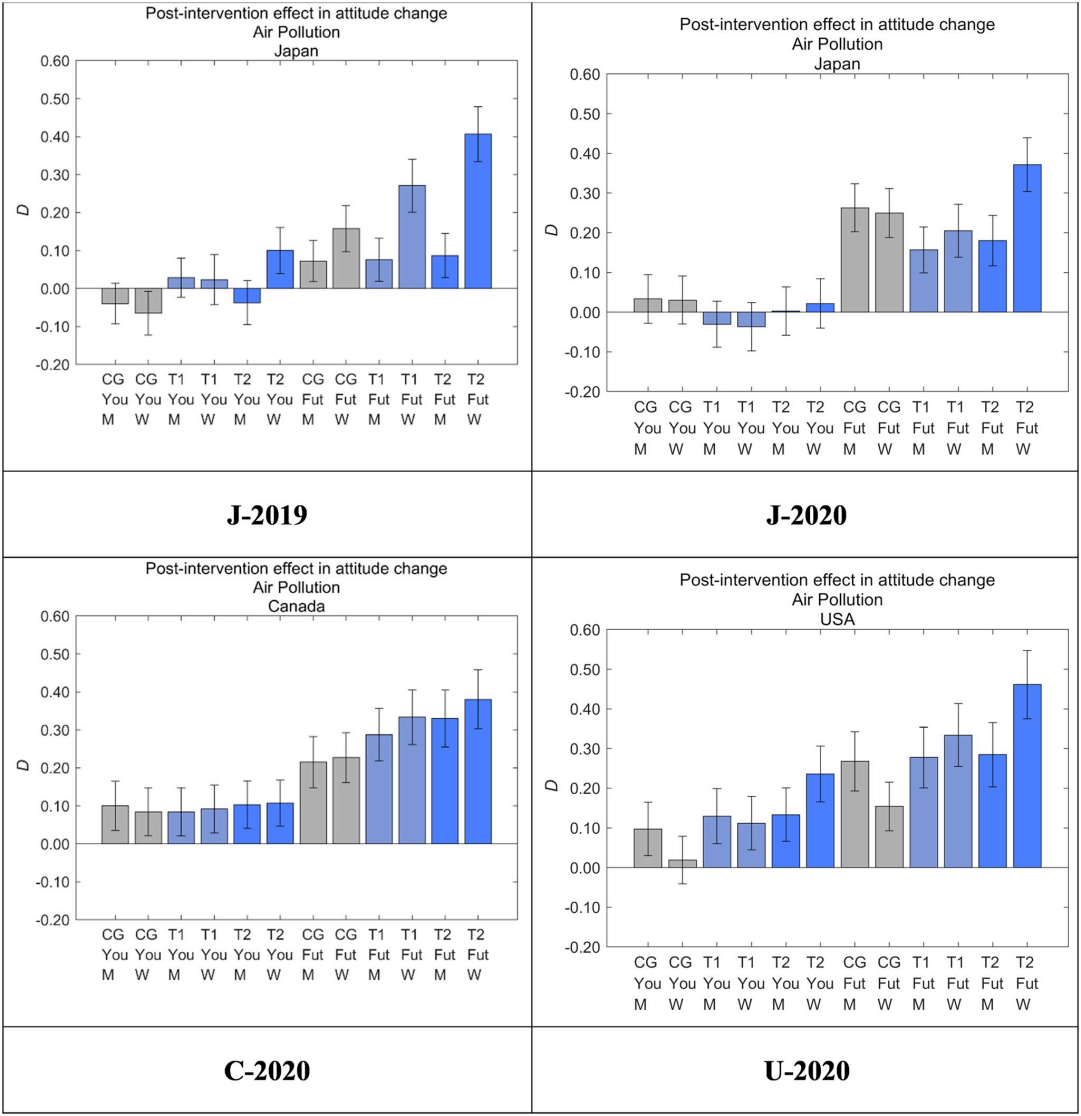
Post-intervention effect in attitude change toward air pollution (*D*) by sex. M, men; W, women. Error bars show 95% confidence intervals. Higher values on the vertical axis indicate lower perceived danger of air pollution.

Fig 12 shows *D* for each dataset and message group by sex, where M represents men and W represents women. In T2 for Future Generations, the effect on women was stronger than that on men in every dataset, and the differences in J-2019, J-2020, and U-2020 were statistically significant. In T2 for Yourself, the effect on women was significantly stronger than that on men only in J-2019, and no significant sex differences were observed in the other message groups in all datasets. Considering the effects for both Future Generations and Yourself, the nudge message in T2 tended to have a stronger effect on women than men, and the degree of the sex differences were greatest in Japan, followed by the US and then Canada. In Canada, the difference between men and women was not statistically significant.

**Fig 13:**
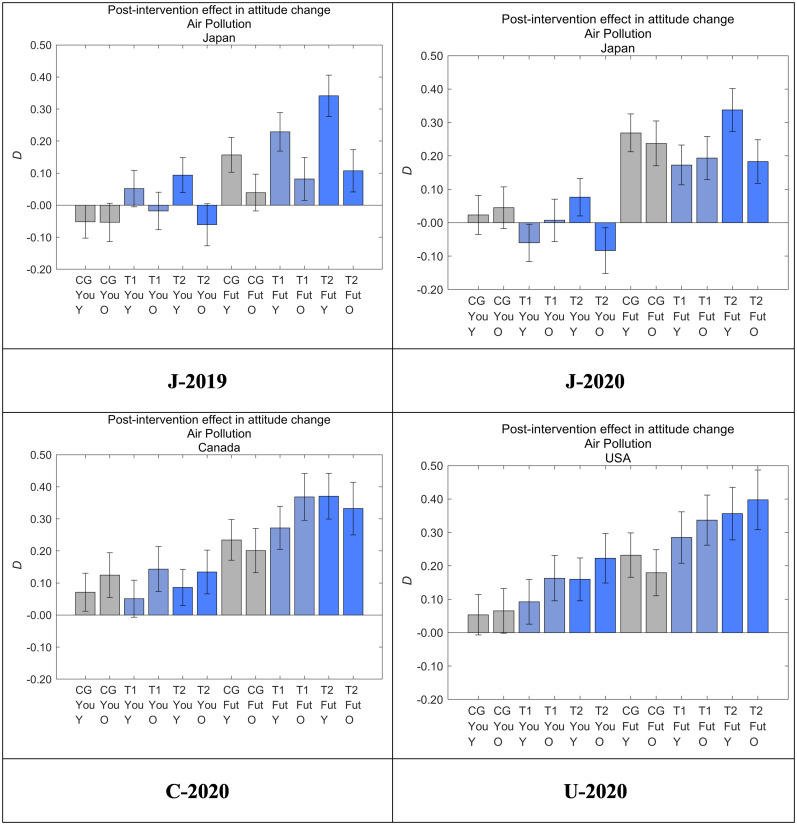
Post-intervention effect in attitude change toward air pollution (*D*) by age. Y, respondents under the age of 50 years; O, respondents 50 years of age and older. Error bars show 95% confidence intervals. Higher values on the vertical axis indicate lower perceived danger of air pollution.

Fig 13 shows *D* for each dataset and message group by age, where Y represents respondents under the age of 50 years and O represents those 50 years of age and older. The message effects were stronger in younger respondents (Y) than in older respondents (O) in T2 for both Future Generations and Yourself in J-2019, and the differences were significant (*p* < 0.001). In J-2020, although the difference was decreased and not significant for Yourself, the difference for Future Generations was still significant. No significant differences between Y and O were observed in C-2020 and U-2020. In these two datasets, the older respondents for Future Generations showed significantly larger intervention effects compared with the two datasets from Japan.

### Panel data analysis

A linear regression model was constructed to quantify the degree to which the nudge message interventions moderated the perceived dangers of air pollution. The model employed dummy variables representing the respondents’ country, the effects of sample attributes, the Big Five traits, and COVID-related variables. The Big Five traits were obtained from the Ten-item Personality Inventory [40, 41]. COVID-related variables are the cumulative numbers of reported cases in per prefecture in Japan, per province in Canada, and per state in the US (Table 2) and the confounding factors multiplied by the dummies for receiving either the T1 or T2 intervention, to adjust the effects of the COVID-19 pandemic, which increased *D* in CG.

$$D=a_{1}\times T1+a_{2}\times T2+a_{3}\times Canada+a_{4}\times US+a_{5}\times S+a_{6}\times A+a_{7}\times P_{ex}+a_{8}\times P_{ag}+a_{9}\times P_{co}+a_{10}\times P_{ne}+a_{11}\times P_{op}+a_{11}\times COVID+a_{12}\times COVID\times TG1+a_{13}\times COVID\times TG2+a_{14}$$ (1)

*T*1, *T*2: Target of the intervention in T1 and T2, respectively (0: no, 1: yes)

*Canada*: Living in Canada (0: no, 1: yes)

*US*: Living in the US (0: no, 1: yes)

*S*: Sex (0: Men; 1: Women)

*A*: Age

*P*_*ex*_: Extraversion

*P*_*ag*_: Agreeableness

*P*_*co*_: Conscientiousness

*P*_*ne*_: Neuroticism

*P*_*op*_: Openness

*COVID*: Number of reported COVID-19 cases in Japan, Canada, and the US

*a*_*1*_–*a*_*13*_: Coefficients for each term

*a*_*14*_: Intercept

Here, *D* is the difference between the answers to *Q*_post_ and *Q*_pre_ for Future Generations or Yourself. To determine *a*_1_–*a*_14_, we performed a forced entry regression by pooling the three datasets for 2020 (J-2020, C-2020, and U-2020) (Table 16). *T*2 was estimated as 0.118 (*p* < 0.001), suggesting that the T2 intervention increased *D* compared with CG and worked as intended. *T2* for Yourself was also significant and the estimate was 0.050 (*p* < 0.01). The estimate for *T*1 (Future Generations) was 0.074 (*p* < 0.01), but it was not significant for Yourself. The results suggest that the T2 intervention for both Future Generations and Yourself had a stronger effect than that for T1. The tendency of a greater effect in T2 compared with T1 and for Future Generations compared with Yourself is consistent with our previous results in Japan [26]. For Future Generations, no statistical significances were observed in the variables *Canada* and *US*. This result suggests that the interventions might be effective independent of culture, at least for Future Generations. Meanwhile, the estimate of *T*2 (Yourself) was 0.093 for *US* (*p* < 0.001) and 0.076 for *Canada* (*p* < 0.01). This suggests that the *T*2 message might have additional intervention effects for Yourself. *S* was estimated as 0.038 (*p* < 0.05), suggesting that the female segment contributed more to *D* than did the male segment. This effect is considered to be caused mainly by the samples in Japan and the US, but not those in Canada where the sex differences in intervention effects were not significant. *A* was estimated as −0.019 (*p* < 0.01), suggesting that young respondents made small but significant contributions to *D* for Future Generations. This age effect is considered to be caused mainly by the samples in Japan, but not those in Canada and the US. For the Big Five traits, *P*_*ag*_ was estimated as 0.022 (*p* < 0.001) for Future Generations and 0.012 (*p* < 0.01) for Yourself, suggesting that high agreeableness, which is related to altruism, contributed significantly to *D* for both Future Generations and Yourself. Although *P*_*ex*_ showed a negative estimate (*p* < 0.05) and *P*_*co*_ showed a positive estimate (*p* < 0.01) for Future Generations, and *P*_*ex*_ for Yourself showed a negative estimate (*p* < 0.1), the contributions were relatively small. The other Big Five traits did not show significant contributions. The two COVID-19 confounding factors tended to be negative for *T*1 (Future Generations) (*p* < 0.05), *T*1 (Yourself), and *T*2 (Future Generations and Yourself) (not significant). Although the statistical significances are not sufficiently clear, they nonetheless suggest that the increase of *D* in CG for J-2020 might have been influenced by the COVID-19 pandemic.

**Table 16:** Coefficients from linear regression analysis using J-2020, C-2020, and U-2020.

Explained variables		Future Generations			Yourself		
		Estimated coefficients	SE	*t*	Estimated coefficients	SE	*t*
**Intercept**		−0.071	0.079	−0.909	−0.074	0.071	−1.043
**Intervention**	T1	**0.074	0.027	2.777	0.006	0.024	0.252
	T2	***0.118	0.026	4.473	*0.050	0.024	2.094
**Country**	Canada	0.016	0.024	0.672	**0.076	0.022	3.459
	US	−0.001	0.026	−0.041	***0.093	0.023	4.044
**Attribute variables**	Sex (Men = 0, Women = 1)	*0.038	0.017	2.160	−0.002	0.016	−0.115
	Age (20s, 30s, 40s, 50s, 60s and older)	**−0.019	0.006	−2.949	0.009	0.006	1.605
**Personality variables**	Extraversion	*−0.007	0.003	−2.039	†−0.005	0.003	−1.731
	Agreeableness	***0.022	0.004	5.181	**0.012	0.004	3.212
	Conscientiousness	*0.010	0.004	2.488	0.000	0.004	−0.022
	Neuroticism	0.001	0.004	0.220	−0.005	0.003	−1.616
	Openness	0.005	0.004	1.326	0.000	0.004	0.102
**COVID-19**	COVID	0.000	0.000	0.423	0.000	0.000	−0.601
	COVID*T1	*−0.069	0.031	−2.237	−0.015	0.028	−0.530
	COVID*T2	−0.027	0.031	−0.877	−0.020	0.028	−0.700
**Adjusted R-squared**		0.007			0.006		
**Number of valid samples**		12385			12385		

^†^, *, **, and *** indicate the difference from zero with 90%, 95%, 99%, and 99.9% confidence, respectively.

### Qualitative survey

After the intervention, the respondents were asked to share their impressions of the messages they received by providing open-ended answers. We received positive and negative responses to this question from all groups, as well as neutral responses, including “none,” “Don’t care,” and “Not sure.” Qualitative analysis of the responses from this section highlights examples of positive, neutral, and negative responses by country that are characteristic of each group (Tables 17–19).

**Table 17:** Typical responses for CG.

	J-2020	C-2020	U-2020
**Positive**	*It would be good if smoke emission from cars could be reduced*. *I hope it could be utilized for preventing global warming*, *not only for preventing health damage*. (Woman in her 40s, *D* = +2 for Yourself, +3 for Future Generations)	*It shows that tech is the cure for the world we live in and will only make the future better* (Man in his 60s, *D* = +2 for Yourself, +4 for Future generations)	*Why has our country not adopted the same type of policies*? *We could have a neutral or even positive impact on the environment and we choose to ignore the facts in front of us*. (Man in his 40s, *D* = +3 for Yourself, +4 for Future Generations)
**Neutral**	*The balance of negative aspects and positive aspects is important*. *Risks are inevitable for industrial improvement to an extent*. (Man in his 60s, *D* = 0 for Yourself, 0 for Future Generations)	*[I’m] not confident the benifit is more than disadvantages* (Man in his 40s, *D* = 0 for Yourself, 0 for Future Generations)	*It’s definitely a trade-off*, *but I consider the positives of modern society and industry being of greater value than the costs associated with it (including pollution)*. *Hopefully*, *we’ll figure out mitigating factors in the future*, *so we can have a modern society without all the negative externalities*. (Man in his 30s, *D* = 0 for Yourself, 0 for Future Generations)
**Negative**	*Everything has become convenient and longevity has been realized*. *But instead*, *we have been facing with more cases of health damage*. *I am not sure it is good or bad*, *I cannot return to the past of inconvenience*. *Though the improvement is proceeding*, *I am scared how the earth itself will be*. (Woman in her 60s, *D* = 0 for Yourself, −1 for Future Generations)	*there is no advantage to pollution it is devastating one point that’s it* (Man in his 40s, *D* = −3 for Yourself, −3 for Future Generations)	*The air pollution was a dangerous situation for human life* (Man in his 30s, *D* = −4 for Yourself, −4 for Future Generations)

Text in italics are the responses as they were written, including typos.

**Table 18:** Typical responses for T1.

	J-2020	C-2020	U-2020
**Positive**	*Different from the past*, *technologies to reduce pollutant emission has been improving and evolving day by day*. *I hope everyday life will be even more convenient*. (Woman in her 40s, *D* = +2 for Yourself, +3 for Future Generations)	*After reading the passage above I have hope for future generations having cleaner air with all the safely precautions that have been set in place and continued improvement*. (Man in his 40s, *D* = +3 for Yourself, +4 for Future Generations)	*It is scary to think of how much our earlier generations dealt with air pollution* (Woman in her 20s, *D* = 0 for Yourself, +4 for Future Generations)
**Neutral**	*Though pollution cannot be prevented for developing new products*, *I think the improvement is necessary for the next generation at the same time*. (Man in his 50s, *D* = +1 for Yourself, 0 for Future Generations)	*its a question of finding the right balance for indutrialization and pollution*, *also investing in future cleaner energy*. (Man in his 50s, D = 0 for Yourself, 0 for Future Generations)	*We don’t live in Japan* (Man in his 60s, *D* = 0 for Yourself, 0 for Future Generations)
**Negative**	*We in this generation should eliminate negative legacy and solve the problem*. (Man in his 60s, *D* = 0 for Yourself, −1 for Future Generations)	*it is hopeful but i dont know why its not hapening in north america*. *i am confused and skeptical there is more to know about this topic* (Woman in her 30s, *D* = −1 for Yourself, −2 for Future Generations)	*The passage makes me think we didn’t know the damage we were doing to the environment generations ago when manufacturing was just getting into high occurrence*. (Man in his 40s, *D* = −2 for Yourself, −4 for Future Generations)

Text in italics are the responses as they were written, including typos.

**Table 19:** Typical responses for T2.

	J-2020	C-2020	U-2020
**Positive**	*Though we tend to focus on negative aspects of industrialization*, *I realized that our life is supported*, *and that measures and regulations improved the influence on environment compared with the past*. (Woman in her 20s, *D* = +1 for Yourself, +3 for Future Generations)	*I like the idea that we get in return what we contribute*. *Therefore all generations are being provided with the necessary things to live because everyone is pitching in*. *It all starts with one group and without prior generations*, *we wouldn’t have the current support and benefits we have now* (Woman in her 20s, *D* = +2 for Yourself, +4 for Future Generations)	*The activities of our grand parents towards industrilization brought several achievements to our generation but more further to this improvement will bring better results to our future generations*. *It was learnt that industrilization will bring tremendous achievement to the society at large but will further contribute to climate change*. *Similarly*, *future generations will have a maximum benefits of those achievements because more research and results will be implemented to address the impact of climate change and air pollution*. (Man in his 40s, *D* = +2 for Yourself, +4 for Future Generations)
**Neutral**	*Easy to understand by the illustration*. *I think elementary school children will easily understand it*. (Woman in her 30s, *D* = 0 for Yourself, 0 for Future Generations)	*Everything is a cycle*. *Industrialization that our parents/grandparents contributed to has led to benefits for our generation and future generations through things like better infrastructure*, *food*, *and medicine*. *When our future generations grow older*, *they contribute and cycle repeats*. (Man in his 30s, *D* = 0 for Yourself, 0 for Future Generations)	*Illustration is easy to understand* (Woman in her 20s, *D* = 0 for Yourself, 0 for Future Generations)
**Negative**	*It is undoubtable that industrialization has been contributed enormously to human life*, *but instead*, *it is also true that the industrialization has also been causing environmental disruption*. *Without any change*, *I feel a little anxious about the future generations’ life*. (Man in his 60s, *D* = −3 for Yourself, −3 for Future Generations)	*I do not agree since the pollution cannot be justified*. (Woman in her 60s, *D* = +1 for Yourself, −2 for Future Generations)	*Honestly I don’t like the hokey pics*. *Takes away from a serous message*. (Woman in her 40s, *D* = +0 for Yourself, −3 for Future Generations)

Text in italics are the responses as they were written, including typos.

For CG, respondents in all the three countries who mentioned benefits expected that industrialization would help to improve the environment. Those who gave neutral responses mentioned the trade-offs or the balance between risks and benefits. Those who mentioned drawbacks focused on pollution itself or the health risks linked to pollution. There was a positive response to Japan’s case in U-2020 despite the respondent living in the US (Table 17).

The overall tendencies of responses for T1 were similar to those for CG, in that positive responses focused on benefits, negative responses focused on risks, and neutral responses mentioned the balance between the two. There were also neutral and negative responses due to the messages mentioning conditions in Japan, which some of the respondents felt had no relevance to themselves (neutral, in U-2020) and even elicited skepticism (negative, in C-2020) (Table 18).

The overall tendencies of responses for T2 were also similar to those for CG and T1, in that positive responses focused on benefits, negative responses focused on risks, and neutral responses mentioned the balance between the two. There were also positive responses to the illustration, which only the T2 message contained, including that it was helpful for understanding the message (neutral, J-2020 and U-2020). Among the negative responses was one from a respondent who felt that the illustration was “hokey” (negative, U-2020) (Table 19).

To investigate how the respondents’ attitudes were affected by the COVID-19 pandemic, we extracted responses from the open-ended answers by using keywords such as “corona,” “covid,” “pandemic,” and “novel coronavirus” in English and Japanese. As a result, 23 respondents in J-2020 and 6 respondents in C-2020 who mentioned COVID-related keywords were found, although no COVID-related keywords appeared in the designed messages or the questionnaire items. There was no response mentioning COVID-related keywords in U-2020. The observed difference in numbers of respondents who mentioned COVID-related words appears to correspond with the fact that the pandemic had already started in Japan whereas the numbers of reported cases in Canada and the US were negligible during the survey term. Respondents who mentioned COVID-related keywords appeared to be negative about industrialization and technological advancement (Table 20).

**Table 20:** Typical responses that mentioned COVID-19.

J-2020	C-2020	U-2020
*We have been benefited very much through the evolution of various things*. *But there are many things that I want to think about*, *such as quick global spread of the new coronavirus caused by travelling people*. (Woman in her 60s, *D* = −1 for Yourself, −1 for Future Generations)	*Industrialization has gotten worse since our parents and grandparents generations*. *More health problems*. *The lakes are being cluttered garbage so is the seas with plastic bottles*. *I believe that more diseases are on the rise*. *Shingles in older people and now the corona virus is a new one*. *The weather is changing getting hotter in the summer months where you can’t even go outside and all the air conditioners are using so much electricity that there will be more power outages*. *The world is changing too many diseases*, *and climate it will be hard for the new generation* (Woman in her 50s, *D* = 0 for Yourself, 0 for Future Generations)	


Text in italics are the responses as they were written, including typographical errors.

## Discussion

Our analysis showed the intervention effects of the designed message interventions were highest in T2, followed by T1 and CG, and were higher for Future Generations than for Yourself. These tendencies were robust across all the three countries, suggesting that our nudge message was effective across cultures. The panel analysis pooling datasets from the three countries revealed that the intervention effect for Future Generations, excluding the respondents’ attribute effects, was 0.118 on a 5-point Likert-type scale, which is about 3% on the whole scale. Considering that the effect size of an existing nudge based on social norms was estimated to be 2.0% [42], the effect size of our nudge can be regarded as being on a similar level, although the approaches and the target variables were different. The adjusted R2 values of 0.007 for Future Generations and 0.006 for Yourself can be regarded as natural, considering the values from a similar type of experiment [26] as well as RCT experiments on nudging information provision using social norms [42].

Some differences in intervention effects per segment and an effect of the COVID-19 pandemic were observed. T2 for Future Generations for C-2020 and U-2020 were higher than for J-2019 and J-2020. Accordingly, post-intervention perceptions of benefiting from the actions of older relatives were higher in C-2020 and U-2020 than in J-2019 and J-2020. The correlations between the message effect sizes and perceptions of benefiting from the actions of older relatives were positive and statistically significant in all the datasets. Thus, the tendency toward perceptions of benefiting from the actions of older relatives contributing to the message effect size to moderate risk-averse attitudes is considered to have universality and is not culturally specific.

The intervention effects were larger in women than in men in most cases. Meanwhile, there was variability in the degree of sex differences in effect sizes, suggesting that the message effect might be universal yet could be modified by cultural factors. In Canada, the sex differences in intervention effects were not significant, whereas the effects were stronger for women than for men in the US and Japan. The existing indexes for sex differences focusing mainly on cultural aspects could be potential explanatory variables for this heterogeneity of responses. There are three known indexes called Gender Development Index (GDI), Gender Inequality Index (GII), and Gender Gap Index (GGI). GDI for 2019 was highest in the US (0.994), followed by Canada (0.986) and Japan (0.978) [43]; GII for 2019 was lowest in Canada (0.080), followed by Japan (0.094) and the US (0.204) [44]; and GGI for 2019 was highest in Canada (0.772), followed by the US (0.724) and Japan (0.652) [45]. Although these three indexes differ according to which aspects are examined, Canada showed sufficiently high scores in all the three indexes compared with the other two countries. Considering that sex differences in the intervention effects were greatest in Japan, followed by the US and Canada, the responses to the nudge messages might be more homogeneous in countries with more homogeneous populations. This possibility suggests that the nudge message might be affected by culture, at least partially, even if the overall effect of the nudge message to elicit perception of benefiting from the actions of older relatives, based on altruism, was consistent and significant in all three countries.

Comparing J-2019 and J-2020, no clear difference was observed in the perception of benefiting from the actions of older relatives and the perception that younger relatives are similarly benefiting (Figs 9 and 10). However, the COVID-19 pandemic was starting to spread at the time J-2020 was being collected. Thus, the decrease of the DID effect on J-2020 compared with J-2019 might possibly have been caused by other factors, such as perceptions of receiving familial support in everyday life, rather than by how the respondents felt about receiving the nudge messages.

The responses to the open-ended question, where positive attitudes toward industrialization was attributed to the benefits and negative attitudes toward air pollution was attributed to the risks, could be interpreted as an interplay between cognitive dissonance [46, 47] and evolutionary science. From an evolutionary perspective, new knowledge usually emerges from modified previous knowledge, where language serves as a tool for accumulating new knowledge separate from emotion [48]. Cognitive dissonance theory suggests that the new knowledge should be discarded, without another tool that mediates separated emotion. In our present study, the positive belief of “Industrialization supports our everyday life.” and the negative belief of “Air pollution might potentially cause health problems.” can contradict each other. Although which belief is valued depends on the respondents’ judgements, the positive descriptions in our designed messages might correspond to the new knowledge. Under this condition, our nudging messages might resolve the cognitive dissonance caused by the two contradicting beliefs, by evoking the positive emotion of being supported by relatives via industrialization, which might consequently contribute to valuing its benefits. For a comparison with previous types of nudges, we considered our framework in the context of salience nudges, in which human attention is drawn to what is novel or seems relevant [49], thereby influencing behavior [50]. Salience nudges have been applied to a wide variety of domains, including food selection [51], waste reduction [52], and vaccinations [53]. We furthermore propose two related subcategories, namely, “pure nudges,” which makes desirable choices easier, and “moral nudges,” which motivates by appealing to emotional responses such as fun, fear, shame, and pride [54]. However, our approach was originally designed based on insights from a simulation model that considered the evolution of altruistic agents. The simulation results showed that agents who were supported by relatives evolved to take risks that are perceived to threaten their younger relatives, even if most of the population evolved to be averse to such risks [21]. This finding led to the current design of our novel nudging messages, which boost the sense of support in order to moderate extreme risk-averse attitudes toward threats. Our framework is unique in that the message design is rooted in an evolutionary simulation model that considers altruism and differs from previous applications of salience nudge or its subcategories, including “pure nudges” or “moral nudges”.

Using another existing taxonomy for nudges, our nudging approach can potentially be interpreted as a “pro-social nudge”, which is aimed at increasing social welfare rather than the decision-maker’s benefit [55]. While the definition of the pro-social nudge is result-oriented in that society should benefit, our approach is motive-oriented in that the intervention acts on altruistic motives. Although results and motives do not always correspond to each other directly, if more types of nudges based on altruistic motives that are similar to our approach are documented in the future, many of them would be expected to function as pro-social nudges. Such pro-social nudges could involve altruism among both concurrent and different generations, including future kin, who sometimes may not be alive when the altruistic behaviors are exhibited. Given that reciprocal altruism is used for inexpensive, continuous, and easily trackable altruism, while kin selection is used for altruism involving high costs and benefits [56], reciprocal altruism is likely to be used among concurrent generations, whereas kin selection–based altruism is likely to be used among different generations. Our designed kin selection–based messages assuming altruism among different generations do not assume the use of responses based on reciprocal altruism. Thus, investigating such reciprocal altruism–based nudging approaches will be a topic of future work, which will require a type of analysis that differs from our approach, such as assessing the signaling effects of displaying altruistic behaviors [14].

Our nudging approach could be potentially interpreted from cognitive-behavioral perspectives [57] too. Given that the risk-averse attitudes toward industrialization can be regarded as a kind of automatic thoughts accompanied by a weak form of anxiety, our designed messages might serve as an intervention that provides counter evidence to relieve anxiety. Furthermore, our nudging messages might prime actual thinking that the attitudes or the consequent actions are rewarding in that their own decisions will benefit future kin.

The importance of altruism in association with kin selection warrants special attention. Before the experimental interventions, respondents were led to perceive risks to future generations as more dangerous to those people than to the respondents themselves. In general terms, support to future generations would originally be performed by the respondents themselves (i.e., the current generation). However, our designed messages conveyed that any cost incurred for supporting future generations would be canceled by the support from previous generations as a form of industrialization. This structure of indirect support from previous generations to future generations (i.e., toward kin in particular) for the current generation possibly caused additional perception of being supported, and hence resulted in a change in their attitudes toward altruism. We measured the perception of benefits (i.e., support) that respondents received from previous generations (see Fig 9), and benefits that past generations gave to future generations (see Fig 10). Furthermore, there was a decrease in perceived risks for future generations (i.e., the target variable), which was strongly correlated with the two kinds of perceived benefits (see Tables 14 and 15). Thus, the value of our work is its clear indication that nudging can occur within the context of kin selection for altruistic behavior.

The message effects of perceived support to future generations potentially have a relationship with episodic future thinking [58], in particular constructive episodic simulation [59], which assumes that people retrieve and recombine elements of past events to simulate future events. Given that the respondents, on reading our designed messages, had positive feelings toward industrialization through which they had been receiving support from their past generations, similar positive feelings might be projected to the relationship between their own generation and future generations. Further, given that episodic future thoughts tend to be positively biased [60], the documented positive feelings could be further strengthened. These dynamics might be another aspect of the perceived support to future generations via industrialization to promote the effects of our designed messages.

Although the nudge messages did not mention COVID-19, the number of open-ended responses mentioning COVID-related keywords was large in J-2020, where the COVID-19 pandemic was just starting to spread. Indeed, the COVID-19 pandemic decreased the DID effect of the nudge message in J-2020. The intervention effects can apparently be affected by exogenous factors such as social events that appear to have no relevance to the designed information. The decrease of the DID effect was caused by an increase of the CG effect as a baseline, rather than the change of the T2 effect. One possible interpretation for this result is that perception of being supported by one’s family in everyday life contributed to the effect size of CG independent of receiving the nudge message intervention, whereas the post-intervention perception of benefiting from the actions of older relatives contributed to the effect size of T2. In fact, although the CG effect for Future Generations was smaller in J-2019 than in the other datasets, the effect size increased in J-2020 and reached the same level as that in C-2020 and U-2020. The samples for J-2020 were collected when many people in Japan began remote working and other aspects of people’s daily lives were changed quickly and drastically due to the COVID-19 pandemic. They started to spend more time at home, in close proximity with their family, compared with before the COVID-19 pandemic, which could explain the increased sense of being supported by one’s family in everyday life. Furthermore, although the number of respondents limits our ability to draw firm conclusions, the nudge effects observed in Japan resulting from COVID-19 would likely have been noticeable in Canada and the US. Considering that the DID effects were weakened in the timing that COVID-19 started to spread, as well as that those who mentioned the COVID-19 tended to show negative attitudes toward industrialization, the nudge effects would decrease in Canada and the US too if the situations caused by the COVID-19 pandemic were similar to Japan.

One limitation of the present study is that the message delivered to the CG may not have been perceived as providing a neutral stance toward industrialization. In hindsight, the benefits from industrialization were unnecessarily emphasized in the message for the CG, which might have been perceived by the participants as being slightly favorable toward industrialization. In the future, researchers may benefit from clearly stating the positive and negative implications of industrialization, or by pre-screening messages to ensure that a neutral stance is perceived. Although the present study led to significant findings, if a less favorable and more neutral position had been used, the effect sizes might have been larger because the DID effects were measured in comparison with the CG. This possibility should be explored in subsequent studies.

## Conclusion

We designed a nudge message based on insights from evolutionary psychological research on altruism and conducted a randomized controlled trial to ascertain its intervention effects. Using over 12,000 samples from Japan, Canada, and the US obtained through an online survey, we showed that risk-averse attitudes toward air pollution resulting from industrialization were significantly moderated by the intervention. The nudge message highlighted benefits resulting from the actions of older generations, as well as the benefits to future generations realized by industrialization. The sense of being supported by one’s family significantly increased the effects of the intervention message.

Although there was variability in the intervention effects per segment, the overall effects of the nudge message intervention showed consistently significant effects in all three countries as expected. The textual information, which explained the relationship of benefiting from the actions of older generations, as well as the benefits to future generations, significantly moderated risk-averse attitudes toward air pollution. By adding an illustration highlighting the relationship of whose actions benefits whom and how, the effect was further boosted. Younger women showed the highest intervention effects. The sex difference in intervention effects was significant in Japan and the US, but not in Canada. For personality traits, higher agreeableness significantly contributed to the intervention effects. Higher conscientiousness and lower extraversion also contributed to the effects, yet the significance was relatively low compared with agreeableness.

One of the most notable and traditional applications of nudges is information provision using social norms, which has been employed to elicit energy conservation behaviors. There have not been any recent significant advances in the application of nudging. Our proposed framework for information provision that considers altruism has the potential to be used for a wide variety of applications that could influence multiple generations of people. An example of this application is information provision regarding new technologies, whose risks are generally perceived to be larger than older technologies. Another potential application is to elicit more specific behaviors to address environmental problems, such as promoting recycling behaviors. In future work, we will investigate the effects of various nudging messages on different topics in order to determine the universality of our proposed framework.

The intervention effect was also affected by the COVID-19 pandemic, even though the designed messages did not mention the virus and the topic had no relevance to the pandemic. The samples from Japan collected at the end of February 2020 showed no significant intervention effects, whereas those from 2019 showed clear and significant effects. The disappearance of intervention effects caused by the COVID-19 pandemic was due to the increased message effect for the control group, rather than decreased effects for the treatment group. This finding was rooted in the increased sense of being supported by one’s family in everyday life, which was considered to be the result of spending more time with one’s family. In this manner, we identified that an event with a large social impact that was unrelated to the nudge message could nevertheless decrease its effects.

Although the nudge message elicited altruism-related responses and was universally effective in all three countries, our analysis revealed that nudging effects can still be affected by exogenous factors such as cultural differences and social contexts. To realize more robust and stable nudging effects, negative effects on interventions caused by exogenous factors should be mitigated. Thus, our future work will investigate how the presented information should be modified according to the cultural background and social situation of those who receive the message. Furthermore, to establish nudges that work not only in the three countries that we investigated in this study but in all countries, it is important to understand how to design nudges that target attributes particular to each country as well as characteristics common to all countries. Thus, our future work will search for culture-specific factors affecting nudge effects by conducting surveys in other countries.

## Supporting information

S1 Dataset (CSV)

S1 File (DOCX)
