# Supplementary material for: Searching for the universality of nudging: A cross-cultural comparison of the information effects of reminding people about familial support
Source: PLoS One. 2022 Nov 22;17(11):e0277969. doi: 10.1371/journal.pone.0277969 (PMC9681120; doi:10.1371/journal.pone.0277969)
Supplement: S1 File — (DOCX) [file pone.0277969.s002.docx]

Supplementary Information for

Searching for the universality of nudging: A cross-cultural comparison of the information effects of reminding people about familial support

Hidenori Komatsu, Hiromi Kubota, Nobuyuki Tanaka, Hirotada Ohashi, Mariah Griffin, Jennifer Link, Glenn Geher, Maryanne L. Fisher

**S1 Questionnaire**

*Questions for interventions*

- *Q*_pre_

How do you think air pollution caused by industrialization affects the following people in daily life?^^[[1]](#footnote-1)^^

|  | **Safe** | **Slightly Safe** | **Neutral** | **Slightly Dangerous** | **Dangerous** |
| --- | --- | --- | --- | --- | --- |
| **Future Generations** | 1 | 2 | 3 | 4 | 5 |
| **Yourself** | 1 | 2 | 3 | 4 | 5 |

- *Q*_post_

(One of the messages for CG, T1, and T2 is shown.**)**

**[Please read the message about industrialization and air pollution and answer the question.]**

Please answer the question below again. How do you think air pollution caused by industrialization affects the following people in daily life?^1^

|  | **Safe** | **Slightly Safe** | **Neutral** | **Slightly Dangerous** | **Dangerous** |
| --- | --- | --- | --- | --- | --- |
| **Future Generations** | 1 | 2 | 3 | 4 | 5 |
| **Yourself** | 1 | 2 | 3 | 4 | 5 |

**S1 Table. Summary Statistics for *Q*_pre_ and *Q*_post_.**

| **Dataset** | **Question** | | **Mean** | **Standard Deviation** | **Min** | **Max** |
| --- | --- | --- | --- | --- | --- | --- |
| **J-2019**  **(*n*=4,062)** | ***Q*_pre_** | **Future Generations** | 3.88 | 1.00 | 1 | 5 |
|  |  | **Yourself** | 3.32 | 1.05 | 1 | 5 |
|  | ***Q*_post_** | **Future Generations** | 3.70 | 1.03 | 1 | 5 |
|  |  | **Yourself** | 3.32 | 0.99 | 1 | 5 |
| **J-2020**  **(n=4,130)** | ***Q*_pre_** | **Future Generations** | 3.85 | 1.01 | 1 | 5 |
|  |  | **Yourself** | 3.19 | 1.04 | 1 | 5 |
|  | ***Q*_post_** | **Future Generations** | 3.61 | 1.05 | 1 | 5 |
|  |  | **Yourself** | 3.18 | 0.97 | 1 | 5 |
| **C-2020**  **(*n*=4,127)** | ***Q*_pre_** | **Future Generations** | 3.96 | 1.15 | 1 | 5 |
|  |  | **Yourself** | 3.14 | 1.24 | 1 | 5 |
|  | ***Q*_post_** | **Future Generations** | 3.66 | 1.22 | 1 | 5 |
|  |  | **Yourself** | 3.05 | 1.21 | 1 | 5 |
| **U-2020**  **(*n*=4,128)** | ***Q*_pre_** | **Future Generations** | 3.72 | 1.29 | 1 | 5 |
|  |  | **Yourself** | 3.06 | 1.31 | 1 | 5 |
|  | ***Q*_post_** | **Future Generations** | 3.42 | 1.36 | 1 | 5 |
|  |  | **Yourself** | 2.94 | 1.27 | 1 | 5 |

*Other questions*

- *Q*1

(One of the messages for CG, T1, and T2 is shown.**)**

Upon reading the message above, do you think you are receiving benefits that increase your health and quality of everyday life from your older relatives, including parents or grandparents?

| **I am benefitting** | **I am benefitting slightly** | **I am not benefitting much** | **I am not**  **benefitting** |
| --- | --- | --- | --- |
| 1 | 2 | 3 | 4 |

- *Q*2

(One of the messages for CG, T1, and T2 is shown.**)**

Upon reading the message above, do you think industrialization is giving benefits of increasing health and quality of everyday life to your younger relatives, including children or grandchildren?

| **It has benefits** | **It has some benefits** | **It has few benefits** | **It does not**  **have benefits** |
| --- | --- | --- | --- |
| 1 | 2 | 3 | 4 |

- *Q*3

(One of the messages for CG, T1, and T2 is shown.**)**

Please let us know your impressions or thoughts when you read the above message.

- *Q*4

Please answer each of the questions below.

#Please answer about your children.

#Please answer 0 for all the questions if you have no children now.

1. How many children do you have?
2. How many children are living with you?
3. How many children are working in a paid job?

- *Q*5

Please let us know your parents’ current status about their living and working. (Please check as many as necessary for each question)

|  | **Father** | **Mother** | **None** |
| --- | --- | --- | --- |
| 1. **Living with you in the same house (or at the same site)** |  |  |  |
| 1. **Working in a paid job** |  |  |  |

- *Q*6

Do you think you are being “supported” by the following people somehow?

“by your family”

| **Supported** | **Supported a little** | **Not supported much** | **Not supported** |
| --- | --- | --- | --- |
| 1 | 2 | 3 | 4 |

**S1 Dataset**

**S1 Dataset. The Data Format for S1_Dataset.csv Describing Responses to *Q*_pre_ and *Q*_post_ per Group and Country.**

| **Group** | **Country** | ***Q*_pre_ for Future Generations** | ***Q*_pre_ for Yourself** | ***Q*_post_ for Future Generations** | ***Q*_post_ for Yourself** |
| --- | --- | --- | --- | --- | --- |
| (CG/T1/T2) | (Japan/Canada/The US) | (1–5) | (1–5) | (1–5) | (1–5) |

**S1 Appendix**

**Table A. Sample Ratios for J-2019 by Region.**

| **Region** | **Estimated area (km^2^)** * | **Collected samples** | |
| --- | --- | --- | --- |
|  |  | **Percentage (%)** | **Counts** |
| **Hokkaido** | 83,424 | 5.1 | 211 |
| **Tohoku** | 66,948 | 6.1 | 251 |
| **Kanto** | 32,434 | 37.3 | 1540 |
| **Chubu** | 66,807 | 17.4 | 717 |
| **Kinki** | 33,126 | 18.6 | 766 |
| **Chugoku** | 31,922 | 5.6 | 233 |
| **Shikoku** | 18,803 | 1.9 | 79 |
| **Kyushu** | 42,231 | 7.9 | 327 |
| **Okinawa** | 2,283 | 5.1 | 211 |

Based on data retrieved from https://www.gsi.go.jp/KOKUJYOHO/MENCHO/backnumber/GSI-menseki20200701.pdf

Estimated area by prefecture and city for 2020. Geospatial Information Authority of Japan. 2019.

Regions are listed from north to south.

**Table B. Sample Ratios for J-2020 by Region.**

| **Region** | **Estimated area (km^2^)** * | **Collected samples** | |
| --- | --- | --- | --- |
|  |  | **Percentage (%)** | **Counts** |
| **Hokkaido** | 83,424 | 4.5 | 186 |
| **Tohoku** | 66,948 | 6.0 | 247 |
| **Kanto** | 32,434 | 38.7 | 1600 |
| **Chubu** | 66,807 | 15.4 | 636 |
| **Kinki** | 33,126 | 20.4 | 841 |
| **Chugoku** | 31,922 | 4.4 | 183 |
| **Shikoku** | 18,803 | 2.3 | 96 |
| **Kyushu** | 42,231 | 7.6 | 314 |
| **Okinawa** | 2,283 | 0.7 | 27 |

Based on data retrieved from https://www.gsi.go.jp/KOKUJYOHO/MENCHO/backnumber/GSI-menseki20200701.pdf

Estimated area by prefecture and city for 2020. Geospatial Information Authority of Japan. 2019.

Regions are listed from north to south.

**Table C. Sample Ratios for C-2020 by Region.**

| **Region** | **Estimated area (km^2^)** * | **Collected samples** | |
| --- | --- | --- | --- |
|  |  | **Percentage (%)** | **Counts** |
| **British Columbia** | 944,735 | 15.0 | 621 |
| **Alberta** | 661,848 | 12.3 | 507 |
| **Saskatchewan** | 651,036 | 3.2 | 134 |
| **Manitoba** | 647,797 | 4.3 | 178 |
| **Ontario** | 1,076,395 | 48.3 | 1995 |
| **Quebec** | 1,542,056 | 8.6 | 355 |
| **New Brunswick** | 72,908 | 2.5 | 104 |
| **Nova Scotia** | 55,284 | 3.7 | 153 |
| **Prince Edward Island** | 5,660 | 0.4 | 18 |
| **Newfoundland and Labrador** | 405,212 | 1.5 | 62 |

Based on data retrieved from https://web.archive.org/web/20110524063547/http://www40.statcan.gc.ca/l01/cst01/phys01-eng.htm Land and freshwater area, by province and territory. Statistics Canada. 2005.

Regions are listed from north to south.

**Table D. Sample Ratios for U-2020 by Region.**

| **Region** | **Estimated area (km^2^)** | **Collected samples** | |
| --- | --- | --- | --- |
|  |  | **Percentage (%)** | **Counts** |
| **Alabama** | 135,767 | 1.8 | 74 |
| **Alaska** | 1,723,337 | 0.2 | 7 |
| **Arizona** | 295,234 | 1.9 | 77 |
| **Arkansas** | 137,732 | 0.9 | 36 |
| **California** | 423,967 | 7.7 | 319 |
| **Colorado** | 269,601 | 1.2 | 49 |
| **Connecticut** | 14,357 | 1.0 | 42 |
| **Delaware** | 6,446 | 0.4 | 17 |
| **District of Columbia (DC)** | 177 | 0.3 | 11 |
| **Florida** | 170,312 | 6.6 | 271 |
| **Georgia** | 153,910 | 3.3 | 136 |
| **Hawaii** | 28,313 | 0.3 | 13 |
| **Idaho** | 216,443 | 0.3 | 14 |
| **Illinois** | 149,995 | 4.0 | 165 |
| **Indiana** | 94,326 | 2.2 | 90 |
| **Iowa** | 145,746 | 1.0 | 42 |
| **Kansas** | 213,100 | 0.8 | 31 |
| **Kentucky** | 104,656 | 2.0 | 81 |
| **Louisiana** | 135,659 | 1.2 | 49 |
| **Maine** | 91,633 | 0.6 | 26 |
| **Maryland** | 32,131 | 2.5 | 102 |
| **Massachusetts** | 27,336 | 2.5 | 104 |
| **Michigan** | 250,487 | 3.4 | 140 |
| **Minnesota** | 225,163 | 1.6 | 64 |
| **Mississippi** | 125,438 | 1.1 | 47 |
| **Missouri** | 180,540 | 2.4 | 99 |
| **Montana** | 380,831 | 0.3 | 14 |
| **Nebraska** | 200,330 | 0.6 | 25 |
| **Nevada** | 286,380 | 1.1 | 47 |
| **New Hampshire** | 24,214 | 0.5 | 22 |
| **New Jersey** | 22,591 | 3.1 | 128 |
| **New Mexico** | 314,917 | 0.3 | 13 |
| **New York** | 141,297 | 7.2 | 298 |
| **North Carolina** | 139,391 | 3.5 | 143 |
| **North Dakota** | 183,108 | 0.2 | 9 |
| **Ohio** | 116,098 | 4.6 | 190 |
| **Oklahoma** | 181,037 | 1.1 | 46 |
| **Oregon** | 254,799 | 1.3 | 55 |
| **Pennsylvania** | 119,280 | 4.8 | 199 |
| **Rhode Island** | 4,001 | 0.5 | 20 |
| **South Carolina** | 82,933 | 1.9 | 80 |
| **South Dakota** | 199,729 | 0.1 | 6 |
| **Tennessee** | 109,153 | 2.5 | 103 |
| **Texas** | 695,662 | 6.7 | 278 |
| **Utah** | 219,882 | 0.8 | 33 |
| **Vermont** | 24,906 | 0.2 | 8 |
| **Virginia** | 110,787 | 2.6 | 106 |
| **Washington** | 184,661 | 1.7 | 70 |
| **West Virginia** | 62,756 | 1.0 | 43 |
| **Wisconsin** | 169,635 | 1.9 | 80 |
| **Wyoming** | 253,335 | 0.1 | 6 |

Based on data retrieved from https://www.census.gov/prod/cen2010/cph-2-1.pdf

United States Summary: 2010, Population and Housing Unit Counts, 2010 Census of Population and Housing. United States Census Bureau. 2012.

Regions are listed alphabetically.

1. The English version of the scale was intended to read “how much do you think” but the word “much” was accidentally omitted. However, no participant raised this issue in their open-ended responses, so we assume that they used the scale appropriately in their responses. [↑](#footnote-ref-1)
